# Tumor- and host-derived heparanase-2 (Hpa2) attenuates tumorigenicity: role of Hpa2 in macrophage polarization and BRD7 nuclear localization

**DOI:** 10.1038/s41419-024-07262-9

**Published:** 2024-12-18

**Authors:** Soaad Soboh, Avital Vorontsova, Malik Farhoud, Uri Barash, Inna Naroditsky, Miriam Gross-Cohen, Marina Weissmann, Yasuhiko Nishioka, Adrian S. Woolf, Neil A. Roberts, Yuval Shaked, Neta Ilan, Israel Vlodavsky

**Affiliations:** 1 Technion Integrated Cancer Center, Rappaport Faculty of Medicine, Technion, Haifa, Israel; 2 Department of Cell Biology and Cancer Science, Rappaport Faculty of Medicine, Technion, Haifa, Israel; 3 Departments of Pathology, Rambam Health Care Campus, Haifa, Israel; 4 Department of Respiratory Medicine and Rheumatology, Tokushima University, Tokushima, Japan; 5 Division of Cell Matrix Biology and Regenerative Medicine, School of Biological Sciences, Faculty of Biology Medicine and Health, University of Manchester, Manchester, UK

**Keywords:** Cancer microenvironment, Cancer microenvironment

## Abstract

Little attention was given to heparanase 2 (Hpa2) over the last two decades, possibly because it lacks a heparan sulfate (HS)-degrading activity typical of heparanase. Emerging results suggest, nonetheless, that Hpa2 plays a role in human pathologies, including cancer progression where it functions as a tumor suppressor. Here, we examined the role of Hpa2 in cervical carcinoma. We report that high levels of Hpa2 correlate with prolonged survival of cervical carcinoma patients. Strong staining intensity of Hpa2 also correlates with low tumor grade. Overexpression of Hpa2 in SiHa cervical carcinoma cells resulted in tumor xenografts that were two-fold smaller than control tumors. Interestingly, even smaller tumor xenografts were developed by SiHa cells overexpressing the Pro140Arg and Asn543Ile Hpa2 missense mutations that were identified in patients diagnosed with urofacial syndrome (UFS). Utilizing the Ras recruitment system, we identified bromodomain-containing protein 7 (BRD7) to interact with Hpa2 and found that both BRD7 and the Hpa2 mutants are translocated to the cell nucleus in tumors developed by the Pro140Arg and Asn543Ile Hpa2 mutants. Utilizing our newly developed conditional Hpa2-KO mice, we further show that Hpa2 plays a critical role in macrophage polarization; in the absence of Hpa2, macrophages are shifted towards pro-tumorigenic, M2 phenotype. Notably, implanting SiHa cervical carcinoma cells together with Hpa2-KO macrophages promoted tumor growth. These results support, and further expand, the notion that Hpa2 functions as a tumor suppressor, co-operating with another tumor suppressor, BRD7.

## Introduction

Heparanase is a unique enzyme due to its endoglycosidase activity, capable of cleaving heparan sulfate (HS) side chains of heparan sulfate proteoglycans (HSPG). The pro-tumorigenic properties of heparanase are well-documented [1–3], and heparanase inhibitors are being evaluated in clinical trials as anti-cancer drugs [4, 5].

*HPSE2*, the gene encoding heparanase 2 (Hpa2), was cloned shortly after the cloning of heparanase, based on sequence homology [6]. Hpa2 lacks, however, intrinsic HS-degrading activity, the hallmark of heparanase, yet retains the capacity to interact with HS in high affinity [7]. Unlike the intense research effort devoted to exploring the significance of heparanase in human diseases, very little attention was given to Hpa2. Hpa2 gained strong attention when two independent research groups reported that the *HPSE2* gene is mutated in a human disease called urofacial syndrome (UFS) [8, 9], mostly resulting in frameshifts that lead to an early stop codon and a truncated protein, leading to Hpa2-null phenotype. This finding clearly implicated Hpa2 in human pathologies. More recently, it was reported that Hpa2 levels are reduced markedly in critically ill COVID patients, in patients diagnosed with sepsis, and in models of kidney diseases and diabetes nephropathy [10–12].

Recent results suggest that Hpa2 is readily detected in normal epithelium whereas its levels are decreased substantially in the resulting carcinomas [13–16], an expression pattern typical of tumor suppressors. Importantly, patients exhibiting high levels of Hpa2 survived longer than patients showing low levels of Hpa2 [7, 15–18]. In addition, overexpression of Hpa2 in cancer cell lines resulted in smaller tumor xenografts, whereas silencing of Hpa2 resulted in bigger tumors [7, 16, 17, 19–22]. These results suggest that Hpa2 is critically important for epithelial cell integrity and functions as a tumor suppressor.

Here, we examined the role of Hpa2 in cervical carcinoma. We report that high levels of Hpa2 correlate with prolonged survival of cervical carcinoma patients. Strong staining intensity of Hpa2 also correlates with low tumor grade. Interestingly, we found that Hpa2 staining intensity in cells that reside in the tumor microenvironment also correlates with lower tumor grade. Given that low tumor grade (i.e., well- to moderate-differentiated tumor cells) is associated with prolonged patient survival [23–25], we concluded that tumor- and host-derived Hpa2 attenuate cervical tumorigenesis. Utilizing our newly developed conditional Hpa2-KO mice [14] we show that Hpa2 plays a critical role in macrophage polarization. We further identified BRD7 as Hpa2-interacting protein and found that attenuation of tumor growth is associated with the nuclear positioning of Hpa2 and BRD7.

## Materials and methods

### Study population

A tissue microarray of 180 cervical carcinoma biopsies (2088a; US Biomax; MD, USA) was subjected to immunostaining, applying anti-Hpa2 antibody (Ab#58) essentially as described [16, 17] and the staining was scored according to the intensity (0: none; +1: weak-moderate; +2: strong) in the malignant cells by an expert senior pathologist in a blinded manner. Hpa2 staining intensity was similarly scored in cells that comprise the tumor microenvironment. Hpa2 staining intensity in the tumor cells and cells in the tumor microenvironment was correlated with the clinical records. The KM plotter service (https://kmplot.com/analysis/) was used to reveal the clinical association between Hpa2 and the survival of cervical carcinoma patients.

### Conditional Hpa2-KO mice

*HPSE2*^fl^ mice (C57BL/6) were described previously [14]. Briefly, for the generation of the *HPSE2* conditional knockout allele (*HPSE2*^fl^), the Neo cassette was flanked by self-deletion anchor (SDA) sites and the cKO region was flanked by loxP sites, directed to the introns flanking exon 5. Homologous recombination was verified via PCR and Southern blot techniques. B6.Cg-Tg (CAG-cre/Esr1*) 5Amc/J mice were purchased from JAX mice (JAX Stock No:004682). These CAGGCre-ERTM transgenic mice have a tamoxifen-inducible Cre-mediated recombination system driven by the chicken beta-actin promoter/enhancer coupled with the cytomegalovirus (CMV) immediate-early enhancer. When bred with mice containing loxP-flanked sequences, tamoxifen-inducible Cre-mediated recombination results in the deletion of the floxed sequences in widespread cells/tissues of the offspring. Cre activation was performed via administration (i.p) of tamoxifen (0.1 ml; Sigma T5648, 20 mg/ml dissolved in corn oil) every day for 5 days, resulting in the removal of exon 5 and the disruption of *HPSE2* open reading frame. Tamoxifen was administrated into 5–6 weeks-old Cre^+^ mice, and to age-matched control C57BL/6 mice (Envigo, Jerusalem, Israel). *HPSE2* gene editing by Cre activation was validated by PCR. For experiments, we utilized 3-to-4-month-old mice (typically 6–8 weeks following the administration of tamoxifen).

### Cells and cell culture

Mouse Lewis lung carcinoma (LLC) cells have been described previously [26]. MDA-MB-231 and MDA-MB-468 human breast carcinoma cells have been described elsewhere [27]. HeLa (HPV 18-positive) and SiHa (HPV 16-positive) human cervical carcinoma cells were purchased from the ATCC. RAW264.7 mouse macrophage cell line was kindly provided by Dr. Moran Benhar (Rappaport Faculty of Medicine, Technion, Haifa, Israel) [28]. Cells were grown in Dulbecco’s modified Eagle’s medium (Biological Industries, Beit Haemek, Israel) supplemented with 10% FCS and antibiotics. SiHa were transfected with a control empty vector (Vo), and the Hpa2 gene construct essentially as described [7]. Cells were similarly transfected with Pro140Arg and Asn543Ile Hpa2 missense mutants that were recently identified in UFS patients [29], selected with G418, expanded and pooled. MDA-MB-231 and MDA-MB-468 cells were similarly transfected with control empty vector (Vo), Hpa2, and Hpa2 gene construct that direct Hpa2 to the cell nucleus vis NLS (Hpa2-Nuc), essentially as described [27]. For xenotransplantation, cells from exponential cultures were detached with trypsin/EDTA, washed with PBS, and brought to a concentration of 5 × 10^7^ cells/ml. Cell suspension (5 × 10^6^/0.1 ml) was inoculated subcutaneously in NOD/SCID mice (*n* = 7). Breast carcinoma cells (2.5 × 10^6^) were inoculated orthotopically into the third mammary fat pad of 8-week-old female NOD/SCID mice (*n* = 7). Xenograft size was determined by externally measuring tumors in 2 dimensions using a caliper. At termination, tumor lesions were removed, weighed, fixed in formalin, and subjected to histological and immunohistochemical analyses essentially as described [17, 19, 21]. Cells were found to be negative for mycoplasma. STR profiling was utilized for the authentication of human cell lines.

### Ras recruitment system (RRS)

In order to identify Hpa2-binding proteins that may mediate the anti-tumorigenic properties of Hpa2, we utilized the Ras recruitment system (RRS) [30, 31]. In this system, a mutant line of yeast (Saccharomyces cerevisiae) will not grow at 37 °C unless myristoylated Ras (mRAS) is recruited to the cell membrane following the interaction of a bait protein with a membrane-localized prey. We fused Hpa2 to mRAS as bait and screened a library of human testis cDNA for potential cDNAs that encode Hpa2-interacting proteins.

### Antibodies and reagents

Anti-Hpa2 #58 polyclonal and 20c5 monoclonal antibodies were described previously [7, 19]. Rat anti-mouse F4/80 antibody was purchased from Serotec; Rat anti-mouse CD31 was purchased from Dianova (Hamburg, Germany). Anti-HIF1α antibody was purchased from Cayman Chemical (Ann Arbor, MI); anti-galectin-3, anti-CAIX, anti-BRD7, and anti-collagen-IV antibodies were purchased from Abcam; Anti-phospho-p38 (Thr180/Tyr182) was purchased from Cell Signaling. Anti-FSP1/S100A4, anti-actin, anti-smooth muscle actin (SMA) monoclonal antibodies and Matrigel were purchased from Sigma (St. Louis, MO). Anti-phospho-Erk (Tyr204), anti-syndecan-1, and anti-Bax antibodies were purchased from Santa Cruz Biotechnology. Anti-CD206, anti-CD36, crystal trace violate (CTV), propidium iodide (PI), and LDH cytotoxicity assay were purchased from BioLegend.

### Real-time PCR analyses

Total RNA was extracted with NucleoSpin RNA extraction kit (Macherey-Nagel; Germany) or TRIzol (Sigma), and RNA (1 µg) was amplified using the one-step PCR amplification kit, according to the manufacturer’s (ABgene, Epsom, UK) instructions. The PCR primer sets utilized in this study are listed in Supplementary Table 1. Data are expressed as the mean level of expression normalized to actin, and data represent the mean ± SEM of triplicate samples [16, 17, 21].

### Macrophage collection and phagocytosis

Mouse peritoneal monocytes/macrophages were harvested from the peritoneal fluid of WT or Hpa2-KO mice 3 days after intraperitoneal injection of thioglycolate (3 ml; 40 mg/ml), essentially as described [26, 32, 33]. Peritoneal exudate cells (5 × 10^6^) were plated in 60-mm dish for 24 h and cultured in Dulbecco’s Modified Eagle’s Medium (DMEM) supplemented with glutamine, pyruvate, antibiotics and 10% fetal calf serum in a humidified atmosphere containing 5% CO_2_ at 37 °C. Non-adherent cells were removed after 24 h by washing and the cells remaining attached were considered macrophages [32]. Macrophage phagocytotic capacity was evaluated using zymosan-coated IncuCyte pHrodo Bioparticles (Essen) according to the manufacturer’s instructions, essentially as described [26]. Briefly, WT and Hpa2-KO macrophages were isolated 3 days after the administration (i.p.) of thioglycolate and plated (2 × 10^4^) on 96-well plates for 24 h. Cells were then washed, and the zymosan-coated fluorogenic bioparticles (5 µL) were added. Once the bioparticles were engulfed by phagocytosis and entered the acidic phagosome, a substantial increase in red fluorescence was observed and monitored by quantitative live cell imaging (IncuCyte ZOOM Live Cell Analysis System; Essen) [26].

### Cell migration, spheroids formation, and cytotoxicity

Migration assay was performed using modified Boyden chambers (6.5 mm in diameter, 8 µm pore size; Corning, Corning, NY), coated with fibronectin (30 µl; 10 µg/ml), essentially as described [22]. Cytotoxicity was estimated by LDH assay performed according to the manufacturer’s (BioLegend) instructions. Spheroids and their interaction with macrophages were carried out essentially as described [34]. Briefly, mouse Lewis lung carcinoma cells (1.5 × 10^4^) were plated in 96 U bottom plates, previously coated with BIOFLOAT FLEX (faCellitate; Germany). Plates were then centrifuged (10 min/300 g) and spheroids were formed after 24 h. WT and Hpa2-KO macrophages were isolated as described above, stained with CTV, and 1 × 10^4^ stained macrophages were co-cultured with individual spheroid for 24 h. Tumor spheroids and attached macrophages were fixed and stained with PI and analyzed by an inverted confocal microscope. For LDH assay, spheroids cultured medium was collected after 4 or 24 h, and cytotoxicity was evaluated.

### Tumorigenicity and immunohistochemistry

Administration of SiHa cells and Co-injection of tumor cells (SiHa) and macrophages was carried out essentially as described [26, 33]. Briefly, SiHa cells were detached with trypsin/EDTA, washed with PBS, brought to a concentration of 5 × 10^7^ cells/ml, and implanted (5 × 10^6^/0.1 ml) subcutaneously (*n* = 7). Macrophages were isolated from WT and Hpa2-Ko mice three days following the administration of Thioglycolate and plated on tissue culture dishes for two days. Cells were then scraped and mixed with SiHa cells at a ratio of 1:1 and cell suspension (10 × 10^6^/0.1 ml) was inoculated subcutaneously at the right flank of NOD/SCID mice (*n* = 7). Xenograft size was determined by externally measuring tumors in 2 dimensions using a caliper. At termination, mice were sacrificed, and tumors were removed and weighed. RNA was extracted from a small portion of the tumor, and the remaining portion was fixed in formalin. Paraffin-embedded 5-µm sections were subjected to immunostaining applying the indicated antibodies using the Envision kit according to the manufacturer’s (Dako) instructions, as described [26, 33]. Pictures were captured with a Nikon Digital Sight camera attached to Nikon Eclipse microscope. For Matrigel experiments, WT and Hpa2-KO macrophages were similarly prepared and were re-suspended in ice-cold Matrigel (3 × 10^6^/ml). 0.5 ml of Matrigel-cell suspension was implanted subcutaneously in WT C57Bl/6 mice (*n* = 6), and Matrigel plugs were excised after 10 days. Matrigel plugs were fixed in formalin, embedded in paraffin, and five-micron sections were subjected to histological evaluation and immunostaining. Matrigel was also implanted in mice without cells as a control. All experiments were performed in accordance with the Technion’s Institutional Animal Care and Use Committee (IL-088-06-2022; OPRR-A5026-01).

### Flow cytometry

WT and Hpa2-KO macrophages were subjected to flow cytometry and were analyzed by Flowjo software, essentially as described previously [35, 36]. The antibodies utilized for flow cytometry analyses are listed in Supplementary Table 2. The cells were also acquired by Time-of-flight mass cytometry (CyTOF) (Fluidigm, South San Francisco, CA, USA) and data were analyzed by Cytobank, as previously described [37].

### Statistics

Results are shown as means ± SE. GraphPad Instat software was used for statistical analysis. The differences between the control and the treatment groups were determined by Student’s *t* test/one-way ANOVA, and post-test analyses were done using Dunnett’s/ Bonferroni multiple comparison test. A value of *P* ≤ 0.05 was considered statistically significant. All experiments were repeated at least three times with similar results.

## Results

### Hpa2 attenuates cervical carcinoma growth

**Fig. 1:**
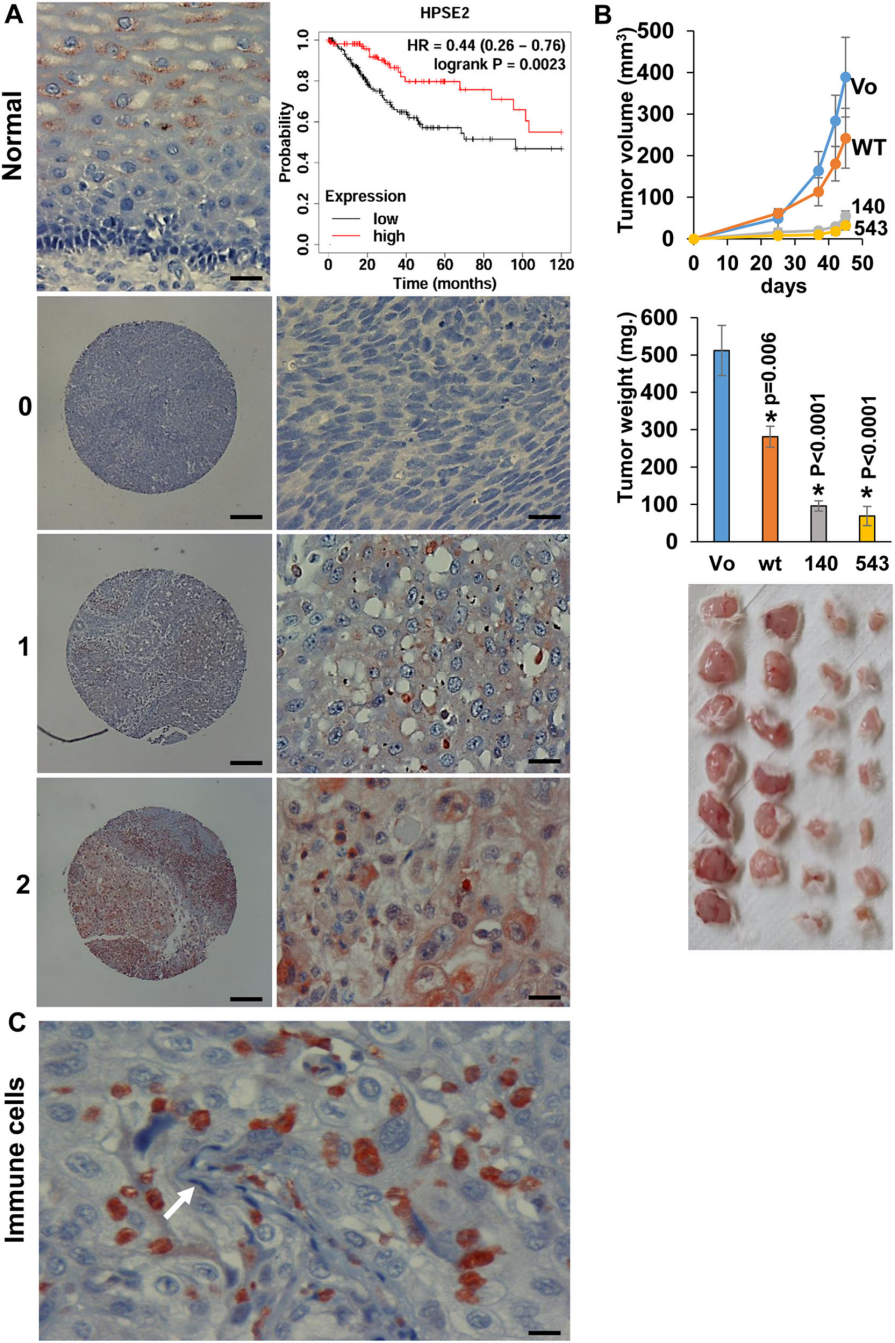
Hpa2 attenuates cervical tumor growth. **A** Immunostaining. Tissue array of 180 biopsies of cervical squamous cell carcinoma and normal cervical tissue were subjected to immunostaining applying anti-Hpa2 antibody (#58). Shown are representative images of Hpa2 staining in normal cervical tissue (upper left panel; scale bars represent 50 microns) and tumor biopsies stained negative (0; second panels) or positive, exhibiting weak (+1; third panels) or strong (+2; fourth panels) staining intensity of Hpa2. Original magnification: ×10 (left panels; scale bars represent 500 microns), ×100 (right panels; scale bars represent 50 microns). Inset. KM survival analysis. Levels of Hpa2 were retrieved from a large cohort (*n* = 304) of cervical carcinoma patients, and survival analysis was calculated by a public server (KM plotter service; https://kmplot.com/analysis). Note prolonged survival of cervical carcinoma patients exhibiting high levels of Hpa2; *P* = 0.002. **B** Tumor growth. SiHa cervical carcinoma cells were transfected with empty vector (Vo), Hpa2 (WT), or with Pro140Arg (140) and Asn543Ile (543) missense Hpa2 mutants. Following selection, cells were expanded, pooled, and implanted (5 × 10^6^) subcutaneously in NOD/SCID mice. Tumor volume was inspected over time using caliper measurements (upper panel). Upon termination, tumors were collected, weighed (second panel), and photographed (third panel). **C** Tumor microenvironment. In addition to Hpa2 immuno-reactivity in tumor cells, the staining also revealed that immune cells that comprise the tumor microenvironment are also stained positive for Hpa2. The white arrow points to fibroblast-like cells that appear Hpa2-negative. Original magnification: ×100 (scale bars represent 50 microns).

Applying the KM plotter service (https://kmplot.com/analysis/) we found that high levels of *HPSE2*, the gene encoding heparanase 2 (*Hpa2*), are associated with prolonged survival of cervical cancer patients (*n* = 304) (Fig. 1, inset). To further reveal the role of Hpa2 in cervical carcinoma we subjected a tissue microarray of 180 cervical squamous cell carcinoma biopsies and normal cervical tissue to immunostaining applying anti-Hpa2 antibody. In normal cervix tissue, Hpa2 staining was detected in the intermediate and superficial cell layers of squamous epithelium (Fig. 1A, left upper panel). In cervical carcinoma we found that 43% (77/178) of the cases were stained negative for Hpa2 while 57% were positive, exhibiting weak (+1; 80/178, 45%) or strong (+2; 21/178, 12%) staining intensity (Fig. 1A and Supplementary Table 3). We next correlated the staining intensity with clinical parameters and found that strong staining of Hpa2 inversely correlates with tumor grade. Hence, most tumors diagnosed as low grade (grade 1 or grade 2) were Hpa2-positive, while 87% of the grade 3 tumors were Hpa2-negative (Table 1; *P* = 0.001). To investigate the molecular mechanism underlying this favorable function of Hpa2 we next transfected SiHa cervical carcinoma cells with Hpa2 gene construct and, following validation of high levels of expression (Supplementary Fig. 1A), cells were inoculated subcutaneously in SCID mice and tumor growth was inspected. Overexpression of Hpa2 resulted in a two-fold decrease in tumor volume (Fig. 1B, upper panel) and weight (Fig. 1B, second panel; *P* = 0.01), further supporting the notion that Hpa2 functions to attenuate tumor growth. Similar inhibition of tumor growth was noted in HeLa cells overexpressing Hpa2 (Supplementary Fig. 1B). Interestingly, even more efficient inhibition of tumor growth was obtained with SiHa cells transfected with the Pro140Arg (hereafter 140) and Asn543Ile (hereafter 543) Hpa2 mutants (Fig. 1B; *P* < 0.0001 and *P* < 0.0001 for 140 vs WT and 543 vs WT, respectively; *P* < 0.001 and *P* < 0.001 for 140 and 543 vs wt, respectively). These two missense mutations were reported to be associated with UFS [29, 38], and were found to exhibit very different biochemical properties. Thus, while the 140 Hpa2 mutant is expressed, secreted, and interact with heparin to extent comparable with WT Hpa2, the 543 Hpa2 mutant is expressed at lower levels, failed to bind heparin, does not get secreted, and was not detected in the cell culture medium even after the addition of heparin [29] (Supplementary Fig. 1C). We have reported recently that in breast carcinoma, unlike other types of cancer, Hpa2 apparently promotes tumor growth and metastasis [27]. Notably, overexpression of the 140 and 543 Hpa2 mutants in MDA-MB-231 breast carcinoma cells promoted tumor weight and lung metastasis to an extent comparable with WT Hpa2 (Supplementary Fig. 2A–D). Increased lung metastasis was also evident in MDA-MB-468 breast carcinoma cells overexpressing WT Hpa2 and the 140 and 543 mutants (Supplementary Fig. 2E). These results support the notion that in cancer, the 140 and 543 mutants are functional to an extent comparable to, or greater than, WT Hpa2.

**Table 1:** Hpa2 staining intensity in the tumor cells correlates inversely with tumor grade.

	Hpa2 staining intensity (Tumor cells)			
	0	1	2	Total
Grade: 1	4 (19)	10 (48)	7 (33)	21
2	29 (27)	64 (60)	14 (13)	107
3	41 (87)	6 (13)	0	47
Total	74	80	21	175^a^

*P* = 0.001.

^a^Data of five cases were missing.

**Fig. 2:**
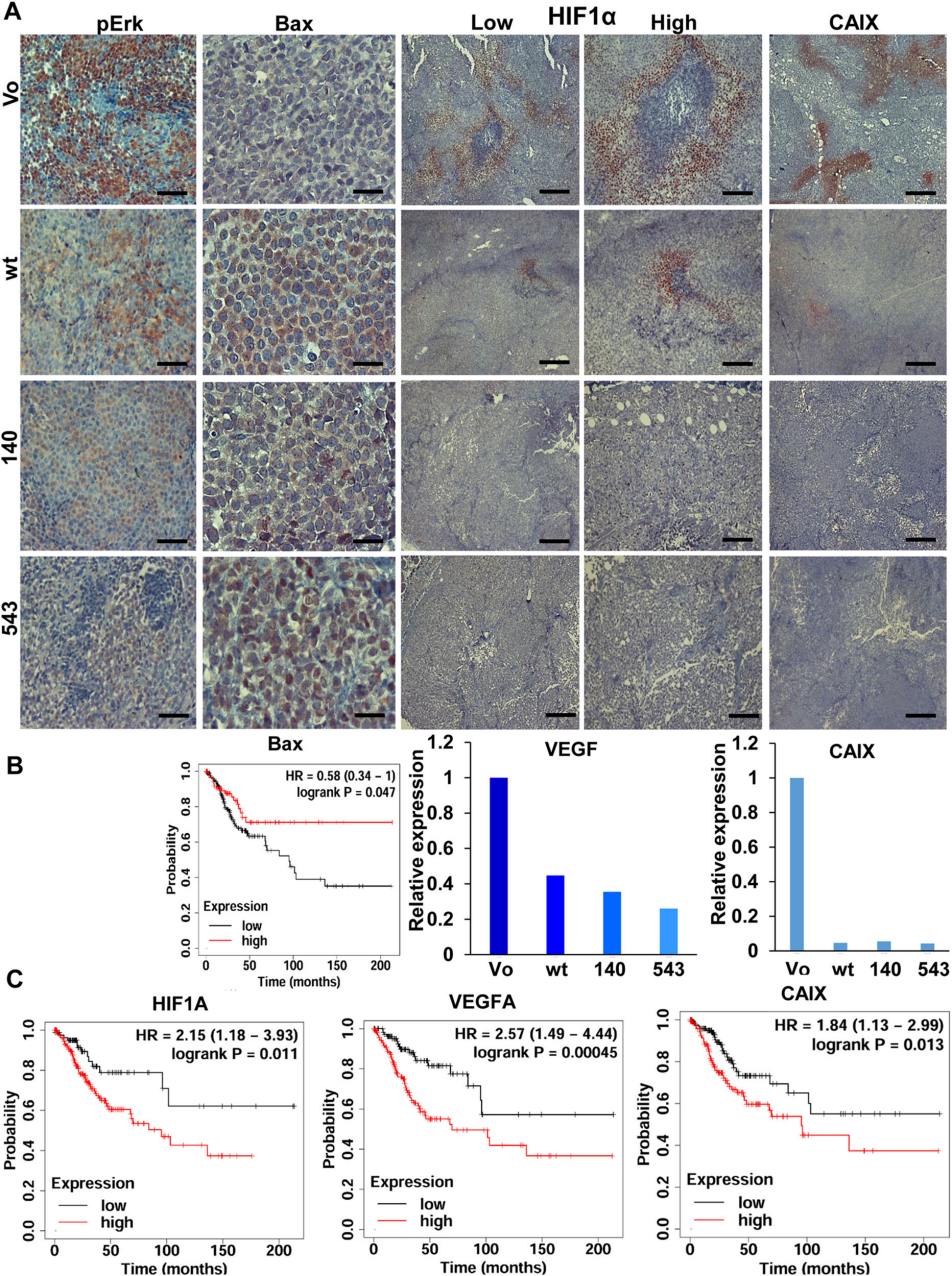
Immunostaining. **A** 5-micron sections of the indicated tumor xenograft were subjected to immunostaining applying antibodies directed against phosphorylated Erk (pErk; left panels), Bax (second left), HIF1α (shown at low and high magnifications; middle and second right panels), and CAIX (right panels). Original magnifications: left, middle, and right panels ×10 (scale bars represent 500 microns); second left panels ×100 (scale bars represent 50 microns); second right panels ×25 (scale bars represent 100 microns). **B**, **C** Levels of Bax, HIF1α, VEGF-A and CAIX were retrieved from a large cohort (*n* = 304) of cervical carcinoma patients and survival analysis was calculated by a public server (KM plotter service; https://kmplot.com/analysis). Note prolonged survival of cervical carcinoma patients exhibiting high levels of Bax (**B**, left panel; *P* = 0.04) and low levels of HIF1α (**C**, left; *P* = 0.01), VEGF-A (**C**, middle; *P* = 0.0004), and CAIX (**C**, right panel; *P* = 0.01). Total RNA was extracted from tumors produced by SiHa control (Vo) and WT Hpa2 or the 140/543 Hpa2 mutants and subjected to qPCR applying primers specific for VEGF-A (**B**, middle) and CAIX (**B**, right panel). Gene expression is shown relative to control (Vo) tumors set arbitrarily to a value of 1 and calculated after normalization to actin.

**Fig. 3:**
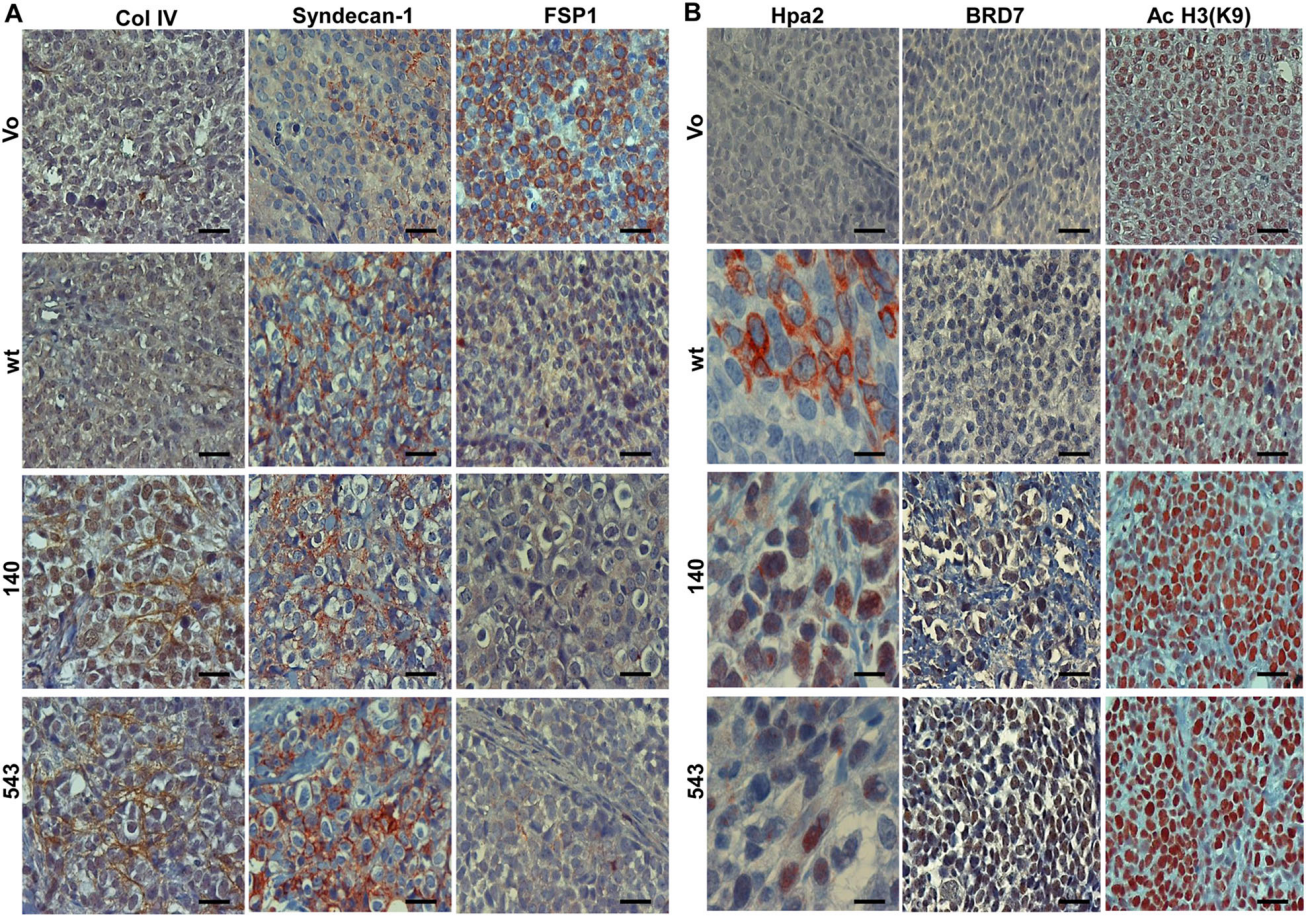
Immunostaining. **A** 5-micron sections of the indicted tumor xenograft were subjected to immunostaining applying antibodies directed against collagen IV (Col IV; left panels), syndecan-1 (second left), and FSP1/S100A4 (FSP1, right). Original magnifications ×100 (scale bars represent 50 microns). **B** Sections were similarly stained for Hpa2 (left panels), BRD7 (middle panels), and histone 3 acetylated at lysine 9 (Ac H3K9; right panels). Original magnifications left panels ×250 (scale bars represent 25 microns); middle and right panels ×100 (scale bars represent 50 microns).

Importantly, we found decreased levels of Erk phosphorylation in SiHa tumors produced by cells overexpressing WT Hpa2 (pErk; Fig. 2A, WT, left panels), and even more prominent decrease in Erk phosphorylation was found in tumors produced by cells overexpressing the 140 and 543 mutants (Fig. 2A, left panels and Supplementary Fig. 3A). Consequently, levels of the pro-apoptotic protein Bax were elevated in Hpa2 tumors and tumors produced by the 140 and 543 mutants (Fig. 2, second left panels and Supplementary Fig. 3B). The 140 and 543 mutants also elicited more efficient tumor fibrosis (Supplementary Fig. 4A, left panels, blue), a feature noted previously for Hpa2 tumors [19]. Furthermore, we found a marked decrease in the staining intensity of hypoxia-inducible factor 1-alpha (HIF1-α) in Hpa2 tumors, and even more substantial decrease was observed in the tumors produced by cells overexpressing 140 and 543 mutants (Fig. 2A, middle and second right panels; Supplementary Fig. 3C). Reduced HIF1-α in Hpa2/140 and 543 mutant tumors was associated with a 2–3-fold decrease in the expression levels of vascular endothelial growth factor-A (VEGF-A), a gene under HIF1α regulation (Fig. 2B, middle panel). Moreover, the staining intensity of carbonic anhydrase IX (CAIX), a HIF1α-inducible gene, was markedly decreased in Hpa2 tumors and tumors produced by cells overexpressing the 140 and 543 mutations (Fig. 2A, right panels). A substantial decrease of CAIX expression by WT and 140/543 mutant Hpa2 was further confirmed by qPCR (Fig. 2B, right) and immunoblotting (Supplementary Fig. 4B). Importantly, low levels of HIF1α, VEGF-A and CAIX are associated with increased survival of cervical carcinoma patient vs patients exhibiting high levels of HIF1α, VEGF-A and CAIX (*n* = 304; Fig. 2C–E). Furthermore, high levels of Bax predict good prognosis for cervical carcinoma patients vs Bax-low patients (Fig. 2B, left). In addition, tumors produced by SiHa cells overexpressing WT Hpa2 and the 140/543 Hpa2 mutants were stained positive for collagen IV (Fig. 3A, left and Supplementary Fig. 3D), suggesting that the cells deposited some basement membrane typical of normal epithelium. Similarly, these tumors exhibited increased levels of syndecan-1 (Fig. 3A, second left, and Supplementary Fig. 3E) that maintain the integrity of epithelial cells [39]. In cervical cancer, high levels of syndecan-1 predict good prognosis (Supplementary Fig. 4C). In contrast, expression of FSP1/S100A4 that promote EMT was decreased substantially in WT Hpa2/140/543 tumors (Fig. 3A, second right and Supplementary Fig. 3F). These results imply that in this tumor model, Hpa2 seemingly functions, to some extent, to promote cellular properties typical of normal epithelium. Given that the 543 mutant does not get secreted and does not bind heparin [29] (Supplementary Fig. 1C), its function in tumorigenesis seems to be executed from within cells. Subjecting sections of the tumor xenografts to immunostaining for Hpa2 revealed that the WT Hpa2 protein exhibits primarily diffuse cytoplasmic staining, likely decorating its biosynthetic route in the ER and Golgi apparatuses (Fig. 3B, WT, left panels), as expected [7]. In striking contrast, the 140 and 543 mutants were noted to localize primarily in the cell nuclei (140, 543; Fig. 3B, left panels), implying that the strong anti-tumorigenic properties of these Hpa2 mutants possibly involve function within the cell nucleus.

In order to reveal Hpa2-associating proteins underlying its anti-tumorigenic properties and, more specifically, its nuclear translocation, we applied the Ras recruitment system (RRS) [30, 31]. To this end, we engineered a mRas-Hpa2 gene construct as bait and screened a membrane-targeted testis cDNA library (prey) for inserts that enable cell growth at the restricted temperature (Supplementary Fig. 4D). Sequencing of these cell colonies repeatedly identified BRD7 to be associated with Hpa2 (Supplementary Table 4). Immunostaining revealed that BRD7 resembles the cellular localization of the 140 and 543 Hpa2 mutants. Hence, very low levels of BRD7 are found in control (Vo) SiHa tumors, while BRD7 levels were predominantly increased in the 140/543 tumors (Fig. 3B, middle panels and Supplementary Fig. 3G). Similarly, a prominent increase in nuclear BRD7 was observed in MDA-MB-231 cells expressing nuclear-targeted Hpa2 (Hpa2-Nuc) vs control tumors (Supplementary Fig. 4E), associating with a prominent attenuation of tumor growth [27]. Since BRD7 interacts with histone 3 once acetylated on lysine 9 and thereby affects chromatin remodeling and gene transcription [40], we next examined histone 3 acetylation levels. In both SiHa and MDA-MB-231 tumor models, nuclear Hpa2/Hpa2 mutants was associated with increased acetylation of histone 3 on lysine 9 (Ac H3K9; Fig. 3B, right panels, Supplementary Fig. 4E, right panels and Supplementary Fig. 4F). This strongly indicates that Hpa2 elicits chromatin modification (acetylation).

### Hpa2 affects macrophage polarization

Careful examination revealed that Hpa2 immunostaining is also detected in cells that comprise the immune aspect of the tumor microenvironment (Fig. 1C), while fibroblasts seem negative for Hpa2 (Fig. 1C, white arrow). We next scored Hpa2 staining in the tumor microenvironment and correlated the staining intensity (Supplementary Table 3) with clinical parameters. Notably, we found that Hpa2 staining intensity in immune cells correlates with lower tumor grade (Table 2; *P* = 0.001). Since low tumor grade (i.e., well-to-moderate-differentiated tumor cells) is associated with prolonged survival of cervical cancer patients [23–25], we concluded that tumor- and host-derived Hpa2 attenuate cervical tumorigenesis.

**Table 2:** Hpa2 staining intensity in immune cells correlates inversely with tumor grade.

	Hpa2 staining intensity (immune cells)			
	0	1	2	Total
Grade: 1	1 (5)	3 (14)	17 (81)	21
2	10 (9)	26 (24)	71 (67)	107
3	39 (83)	5 (11)	3 (6)	47
Total	50	34	91	175^a^

*P* = 0.001.

^a^Data of five cases were missing.

### Hpa2 plays a role in macrophage polarization

To explore the role of Hpa2 in immune cells we utilized a conditional Hpa2-KO mouse. In this newly developed mouse strain [14], exon 5 of *HPSE2* is excised by tamoxifen-induced Cre recombination, resulting in Hpa2-null genotype. We used a transgenic mouse in which expression of the Cre recombinase is driven by the chicken β-actin promoter, thus directing high levels of expression in essentially all cells and tissues [14]. We first collected bone marrow cells from WT and Hpa2-KO mice and the abundance of immune cells was determined by mass cytometry (Cytof) methodology. The results showed that the number of NK, CD4-positive T cells, B cells, and dendritic cells is reduced, whereas the monocyte number is increased in Hpa2-KO vs WT marrow (Supplementary Fig. 5A, B). To confirm these results, we subjected bone marrow cells to FACS analysis. The analyses revealed a significant decrease in the percentage of MHC-II-positive myeloid cells (dendritic cells and macrophages; *P* = 0.004), NK cells (*P* = 0.01), B cells (CD19-positive; *P* = 0.03), and CD4-positive T cells (*P* = 0.005) in Hpa2-KO vs WT bone marrow (Supplementary Fig. 5C), while the abundance of monocytes (Ly6C-positive) was increased (Supplementary Fig. 5C, right; *P* = 0.01). We next employed a similar approach and examined the abundance of immune cells in the spleen of WT and Hpa2-KO mice. Cytof analysis showed that the abundance of progenitor B cells decreased markedly in the spleen of Hpa2-KO vs WT mice, whereas the abundance of neutrophils and MHC-II-positive dendritic cells was increased (Supplementary Fig. 6A, B). FACS analyses confirmed and further expanded these results. A significant decrease in the percentage of NK cells was quantified in Hpa2-KO vs WT spleen (*P* = 0.01), whereas the abundance of myeloid cells (*P* = 0.03), macrophages (*P* = 0.01), CD11b^**+**^/F4/80^**+**^/CD11c^+^ (M1) macrophages (*P* = 0.03), and MHC-II-positive macrophages (*P* = 0.02) was significantly increased in Hpa2-KO vs WT spleen (Supplementary Fig. 6C).

**Fig. 4:**
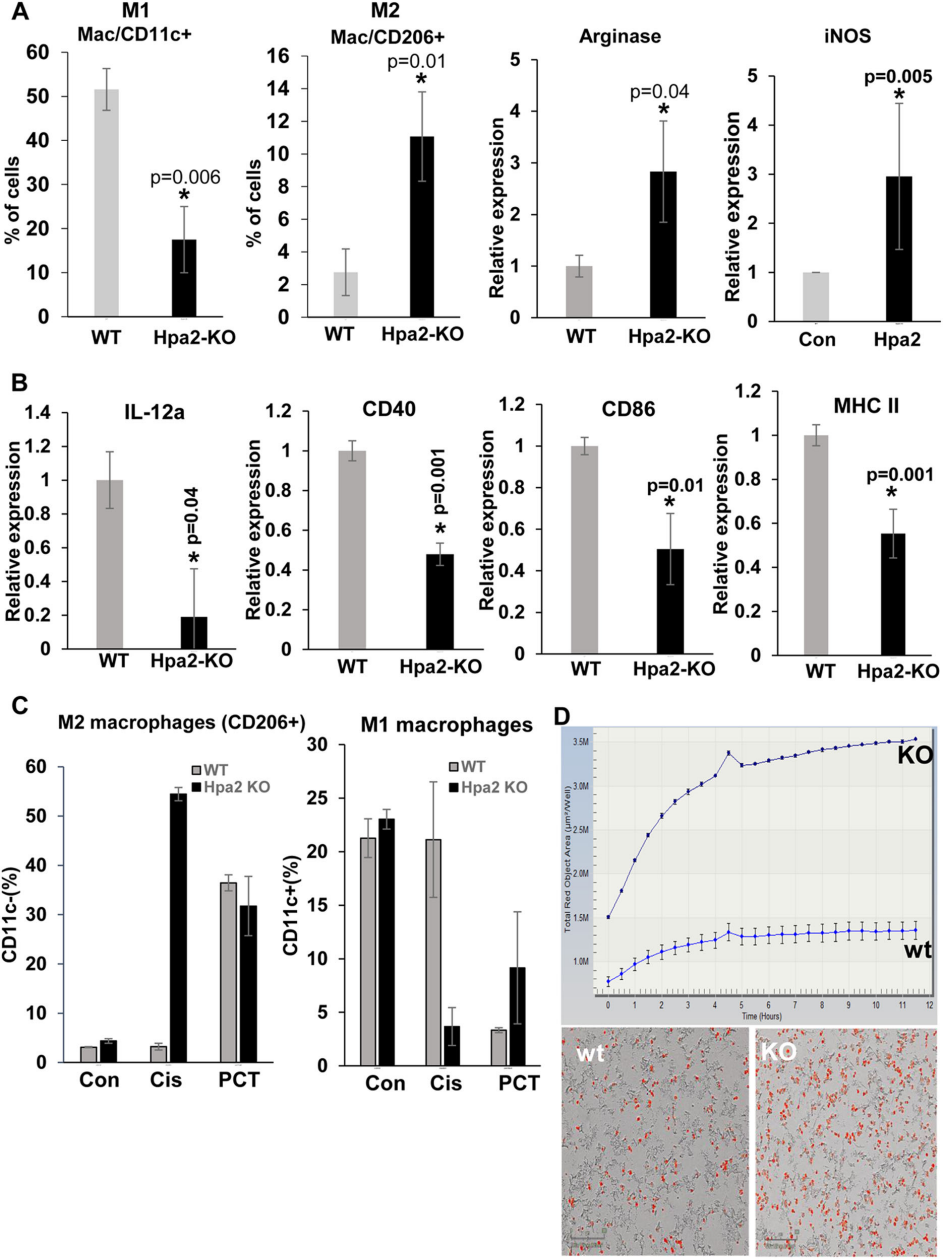
Hpa2-KO macrophages are shifted towards M2 phenotype. **A** M1 and M2 markers. WT and Hpa2-KO mice (*n* = 12) were administrated with thioglycolate and peritoneal fluid was collected three days later. Cells were subjected to FACS analyses, employing cell surface markers typical of M1 (CD11c + ) and M2 (CD206 + ) macrophages (left panels). Total RNA was extracted from corresponding cells and subjected to qPCR analysis applying primers specific for arginase, typical for M2 macrophages (second right). Purified recombinant Hpa2 protein (1 ug/ml) was added exogenously to Hpa2-KO macrophages, total RNA was extracted after 24 h and was subjected to qPCR applying primers specific for iNOS, typical of M1 macrophages (right panel). **B** M1 markers. Total RNA was extracted from WT and Hpa2-KO macrophages and was subjected to qPCR analyses applying primers specific for IL-12a, CD40, CD86, and MHC-II, typical for M1 macrophages. **C** Chemotherapy. WT and Hpa2-KO macrophages were left untreated (Con) or were treated with cisplatin (Cis; 10 μg/ml) or paclitaxel (PCT; 15 μg/ml). After 24 h cells were collected and subjected to FACS analyses employing M2 (CD206^**+**^/ CD11C^**-**^) and M1 (CD11C^**+**^) markers. **D** Phagocytic assay. Macrophages were collected from thioglycolate-treated WT and Hpa2-KO mice (*n* = 5), plated on fibronectin-coated 96-well dish (2 × 10^4^) for 24 h and, following washes, Zymosan-coated fluorogenic bioparticles (5 µl) were added. Macrophage phagocytosis capacity was quantified over time using IncuCyte methodology. Shown are color intensity over time (upper panel) and representative images of phagocytotic macrophages (lower panels, red). Note the increased phagocytic capacity of Hpa2-KO macrophages, typical for M2.

**Fig. 5:**
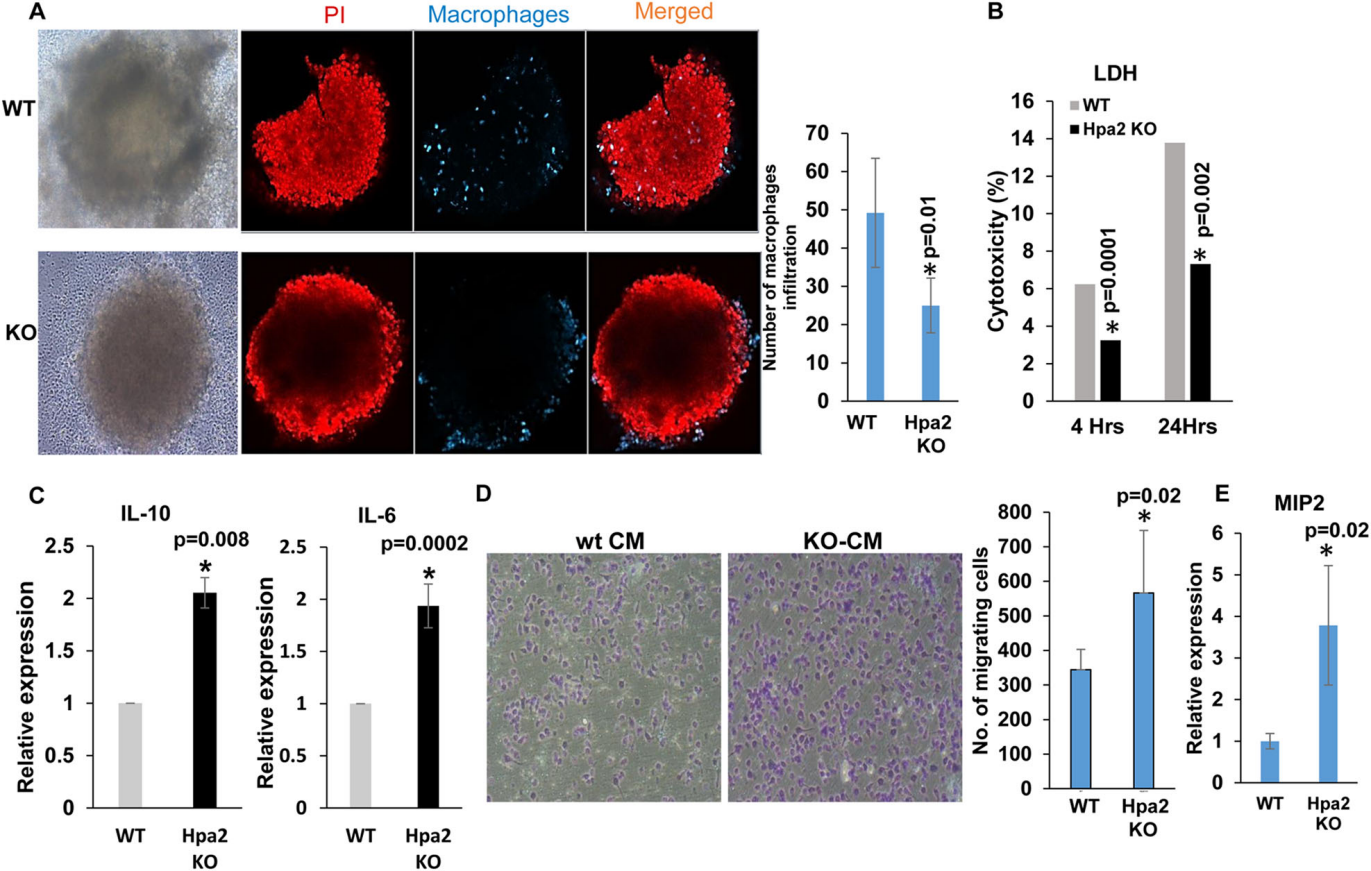
Hpa2-KO macrophages fail to populate tumor spheroids. **A** Macrophages were isolated from thioglycolate-treated WT and Hpa2-KO mice, labeled with Crystal Trace Violet (CTV) and were added (1 × 10^5^) to pre-established spheroids of LLC cells. Spheroids were collected 24 h thereafter, and the presence of macrophages within tumor spheroids was examined by microscopy (**A**, left panels) and quantified (**A**, right panel). Shown are images captured by light (×10; left) and confocal microscopy (×10; z-stack; right). Note that Hpa2-KO macrophages fail to penetrate the tumor spheroids. **B** LDH activity. Conditioned medium was collected after 4 and 24 from corresponding cultures and LDH activity, indicative of cytotoxic activity, was quantified. Note that Hpa2-KO macrophages failed to populate tumor spheroids and exhibited reduced cytotoxic activity typical of M1 macrophages. **C** qPCR. Total RNA was extracted from WT and Hpa2-KO macrophages and was subjected to qPCR analyses applying primers sets specific for IL-10 and IL-6. Cytokine expression by Hpa2-KO macrophages is presented in relation to WT macrophages, set arbitrarily to a value of 1, and normalized to the expression of actin. Note increased expression of pro-tumorigenic IL-10 and IL-6 by Hpa2-KO macrophages. **D** Cell migration. Medium conditioned by WT and Hpa2-KO macrophages was used as a chemoattractant in the Boyden chamber migration assay of RAW264.7 mouse macrophage cell line. Representative images of the migration assay are shown in the left panels; Quantification of cell migration is shown graphically in the right panel. **E** MIP2 expression. Total RNA was extracted from WT and Hpa2-KO macrophages and was subjected to qPCR analysis applying primers specific for MIP2. MIP2 expression in Hpa2-KO macrophages is presented relative to WT macrophages, set arbitrarily to a value of 1, and after normalization to the expression of actin.

To stimulate inflammation, we next employed thioglycolate as an irritant and examined the abundance of immune cells in the spleen and peritoneal fluids following peritonitis. Notably, this relatively mild stimulus elicited fourfold to eightfold increase in the percentage of monocytes (Ly6C-positive; *P* = 0.01), myeloid cells (*P* = 0.01), neutrophils (LY6G-positive; *P* = 0.03), macrophages (*P* = 0.03), and CD11c-positive (M1) macrophages (*P* = 0.05) in Hpa2-KO vs WT spleen (Supplementary Fig. 6D). Importantly, we found that Hpa2 deficiency resulted in a decreased abundance of CD11c-positive (M1) macrophages (*P* = 0.006), and increased percentage of M2 (CD206-positive; *P* = 0.01) macrophages (Fig. 4A, left panels) in the peritoneal fluid. This agrees with the increased expression of arginase (Fig. 4A, second right; *P* = 0.04), a marker for M2 macrophages. Furthermore, the exogenous addition of purified Hpa2 to macrophages isolated from Hpa2-KO mice stimulated the expression of iNOS (Fig. 4A, right panel; *P* = 0.005), a marker for M1 macrophages. A shift toward M2 macrophage polarization in the absence of Hpa2 is also supported by decreased expression of IL-12a (*P* = 0.04), CD40 (*P* = 0.001), CD86 (*P* = 0.01) and MHC-II (*P* = 0.001; Fig. 4B), typical of M1 macrophages [41]. Interestingly, M2 polarization was markedly increased following treatment of Hpa2-KO macrophages with cisplatin, accompanied by a comparable decrease in M1 features (Fig. 4C, Cis), suggesting that in the absence of Hpa2, cisplatin chemotherapeutics may fail. In striking contrast, the polarization of WT macrophages was not affected by cisplatin (Fig. 4C). Treatment with paclitaxel did not show such an effect (Fig. 4C, PCT). In addition, Hpa2-KO macrophages exhibited a prominent increase in phagocytic activity typical of M2 macrophages (Fig. 4D). We next established spheroids of LLC cells [34] and found decreased recruitment of Hpa2-KO macrophages to these tumor spheroids (*P* = 0.01; Fig. 5A, right panel), along with reduced cytotoxicity (Fig. 5B), a feature typical for M1 macrophages. Collectively, these results strongly imply that Hpa2 is critically important for macrophage polarization; in the absence of Hpa2, macrophages exhibit decreased pro-inflammatory (M1) properties and acquire a pro-tumorigenic, M2 phenotype. Moreover, we found that the expression of IL-10 (*P* = 0.03) and IL-6 (*P* = 0.002) is increased by Hpa2-KO macrophages (Fig. 5C), cytokines that can potentially promote tumorigenesis [42]. In agreement with the induction of cytokines expression, we found that medium conditioned by Hpa2-KO macrophages is far more efficient as chemoattractant than medium conditioned by WT macrophages, resulting in increased migration of RAW264.7 macrophage cells (*P* = 0.02; Fig. 5D, right panel), associated with a fourfold increase in MIP2 expression (Fig. 5E; *P* = 0.02).

**Fig. 6:**
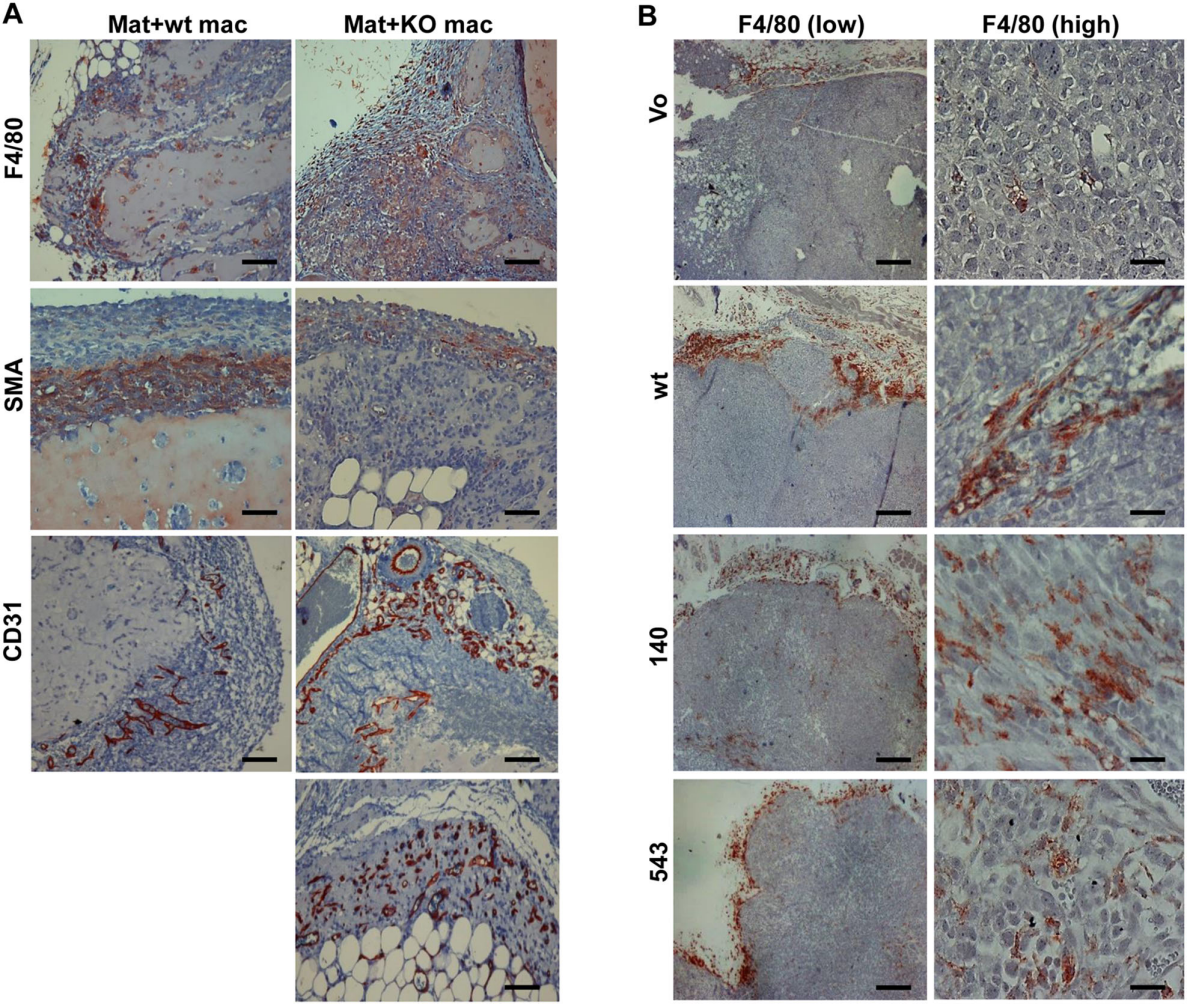
Hpa2-KO macrophages exert pro-tumorigenic properties. **A** Matrigel plug. Macrophages were isolated from thioglycolate-treated WT and Hpa2-KO mice and were suspended in Matrigel (3 × 10^6^/ml). Matrigel containing WT (Mat+WT) or Hpa2-KO (Mat+KO) macrophages was injected subcutaneously (0.5 ml/mouse) in C57Bl/6 mice; Matrigel plugs were collected 10 days thereafter, fixed in formalin, and embedded in paraffin. 5-micron sections were subjected to immunostaining applying anti-F4/80 (upper panels), anti-SMA (second panels), and anti-CD31 (third and fourth panels) antibodies. Original magnifications: ×10 (scale bars represent 500 microns); ×100 (lower right panel; scale bars represent 50 microns). **B** Immunostaining. 5-micron sections of the indicted tumor xenograft were subjected to immunostaining by applying antibody directed against F4/80 (a marker for macrophages) shown at low (×10, left panels; scale bars represent 500 microns) and high (×100, right panels; scale bars represent 50 microns) magnifications. Note increased recruitment of macrophages by tumors produced by cells overexpressing Hpa2 and Hpa2 mutants.

**Fig. 7:**
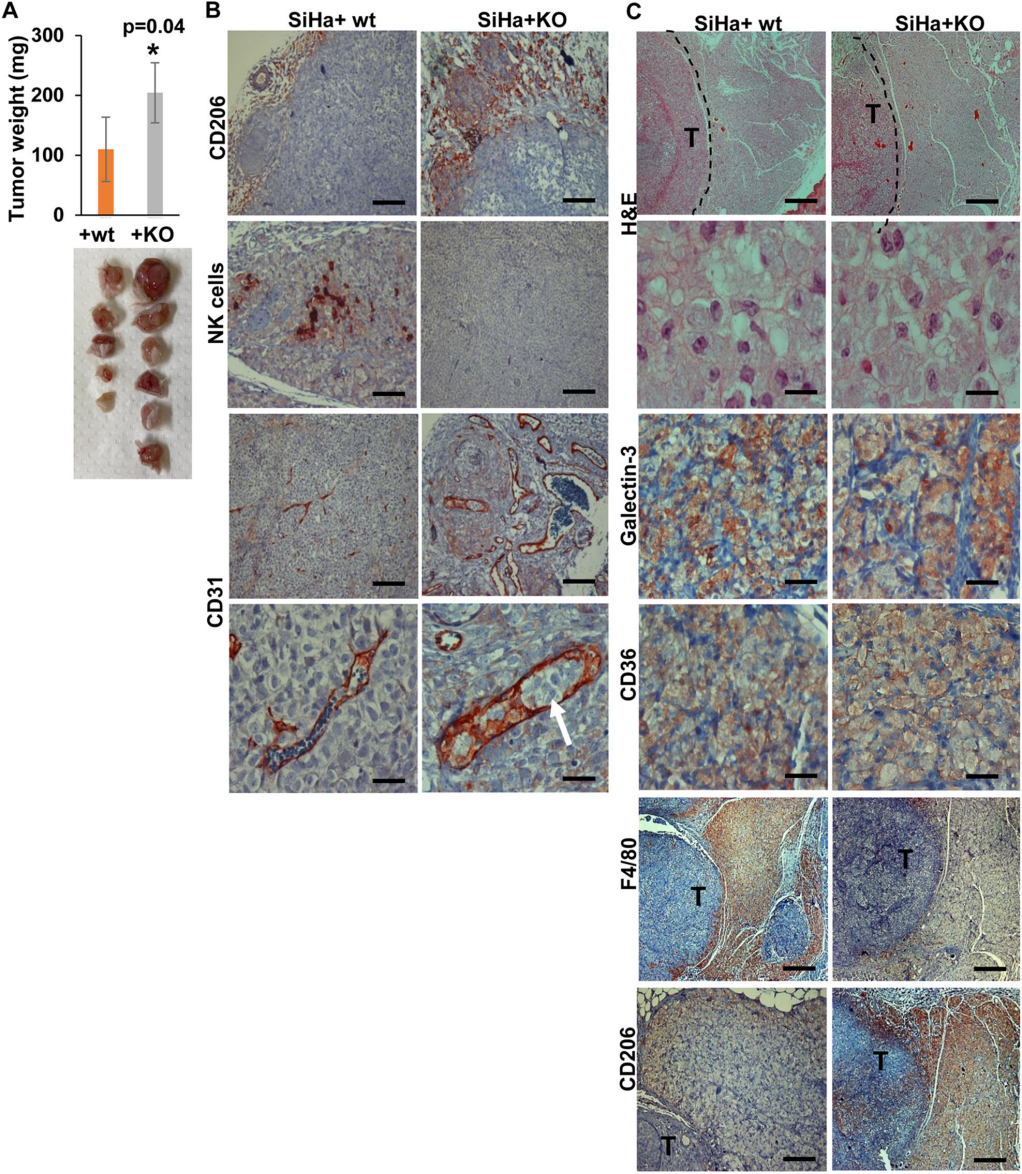
Hpa2-KO macrophages promote tumor growth. **A** Co-implantation. Tumor growth. SiHa cervical carcinoma cells (5 × 10^6^) were mixed with an equal number of WT or Hpa2-KO macrophages and the cell suspension was inoculated subcutaneously in NOD/SCID mice (*n* = 5–6). At termination, tumors were excised, weighed (upper panel) and photographed (lower panel). Note that Hpa2-KO macrophages facilitate tumor growth. **B** Immunostaining. 5-micron sections of the indicted tumor xenograft were subjected to immunostaining applying antibodies directed against CD206 (upper panels), NK1.1 (a marker of NK cells; second panels), and CD31 (a marker of vascular endothelial cells; lower panels). Note increased tumor vascularity and intravasation of tumor cells to the vasculature of tumors produced by SiHa+Hpa2-KO macrophages. Original magnifications: ×100 (upper, second and lower panels; scale bars represent 50 microns); ×25 (third panel; scale bars represent 200 microns). **C** Accumulation of macrophages adjacent to tumor lesions. 5-micron sections of the indicted tumor xenograft were subjected to Hematoxylin & Eosin staining (H&E) shown at low (×10; upper panels; scale bars represent 500 microns) and high (×100; second panels; scale bars represent 50 microns) magnifications. Note the accumulation of foam-like cells (macrophages) adjacent to the tumor (T) lesion. Sections were also subjected to immunostaining applying antibodies directed against galectin-3 (third panels), CD36 (fourth panels), F4/80 (fifth panels) and CD206 (lower panels). Original magnification: third and fourth panels: ×100 (scale bars represent 50 microns), lower panels: ×10 (scale bars represent 500 microns).

To examine the biological significance of the seemingly different macrophage sub-populations we collected peritoneal fluids from WT and Hpa2-KO mice following the administration of thioglycolate, mixed the cells with semi-solid basement membrane (Matrigel), and implanted the Matrigel subcutaneously in C57Bl/6 mice. Notably, Matrigel implanted with Hpa2-KO macrophages was found to recruit far more macrophages from the host (F4/80; Fig. 6A, upper panels), whereas the recruitment of smooth muscle actin (SMA)-positive cells (i.e., fibroblasts) was reduced (Fig. 6A, second panels). Moreover, Matrigel plugs implanted with Hpa2-KO macrophages appeared more vascularized (CD31), and more blood vessels were detected adjacent to the plug (Fig. 6A, third panels) and inside the plug (Fig. 6A, lower panel). These results suggest that increased expression of cytokines by Hpa2-KO macrophages (Fig. 5C) resulted in the recruitment of host (M2) macrophages to the plug, leading to a strong angiogenic response. To examine this possibility in the context of tumor growth, we first subjected SiHa tumors to immunostaining. We found that more macrophages are recruited to WT Hpa2 and the 140/543 mutant tumors vs control (Vo) tumors, localizing at the tumor periphery (Fig. 6B, left) and the center of the tumor (Fig. 6B, right). We next isolated macrophages from WT and Hpa2-KO mice, mixed them with SiHa cells (1:1 ratio) and the mixed cell population was implanted subcutaneously in SCID mice. Notably, SiHa cells implanted with Hpa2-KO macrophages developed bigger tumors vs SiHa cells implanted with WT macrophages (Fig. 7A). Moreover, increased tumor growth in the presence of Hpa2-KO macrophages was associated with extensive staining of CD206 that mark M2 macrophages (Fig. 7B, upper panels), whereas decreased tumor growth in the presence of WT macrophages was associated with the recruitment of NK cells that were not detected in SiHa+Hpa2-KO tumors (Fig. 7B, second panels). Moreover, once co-implanted with KO macrophages, SiHa tumors appeared more vascularized (Fig. 7B, third panels) and tumor cells were readily detected within the lumen of CD31-positive vessels (Fig. 7B, fourth panels, arrow). Such intravasation of tumor cells was not evident in tumors produced by SiHa+WT macrophages (Fig. 7B, fourth left panel) or SiHa cells alone. These results strongly imply that the M2 nature of Hpa2-KO macrophages, increased cytokine expression, and pro-angiogenic capacity promote tumor growth. Intriguingly, we observed a massive accumulation of cells adjacent to the tumor lesions (T; Fig. 7C, upper panels). At higher magnification, these cells exhibited a morphology that resembled foam cells. Indeed, these cells were stained positive for galectin-3 and CD36 (Fig. 7C, third and fourth panels, respectively), markers typical of foam cells [43, 44], and were stained positive for F4/80 (Fig. 7C, fifth left panel). Interestingly, these foam-like cells adjacent to tumors produced by SiHa+Hpa2-KO macrophages appeared F4/80-negative (Fig. 7C, fifth panel, right) but CD206-positive (Fig. 7C, lower panel, right). This possibly implies that due to its secreted nature, Hpa2 originating from macrophages affects not only cells in the tumor microenvironment but also the cells adjacent to the tumor mass.

## Discussion

Here, we examined the role of Hpa2 in cervical cancer, the fourth most frequently diagnosed cancer and the fourth leading cause of cancer death in women, with an estimated 604,000 new cases and 342,000 deaths worldwide in 2020 [45]. We report that cervical cancer patients exhibiting high levels of Hpa2 survive longer than Hpa2-low patients do. Moreover, overexpression of Hpa2 in cervical carcinoma cells attenuates tumor growth, thus supporting the notion that Hpa2 functions as a tumor suppressor. We also found that Hpa2 missense mutations that were recognized in patients with UFS and predicted to result in Hpa2-null phenotype, are functional in tumorigenesis to an extent similar to or greater than WT Hpa2. Importantly, we found that Hpa2 originating from cells that comprise the tumor microenvironment also exerts anti-tumorigenic properties, thus expanding the scope of Hpa2 in tumorigenesis. Utilizing our newly developed conditional Hpa2-KO mouse [14] we report, for the first time, that Hpa2 plays a critical role in macrophage polarization.

### A role of Hpa2 in cervical cancer

**Fig. 8:**
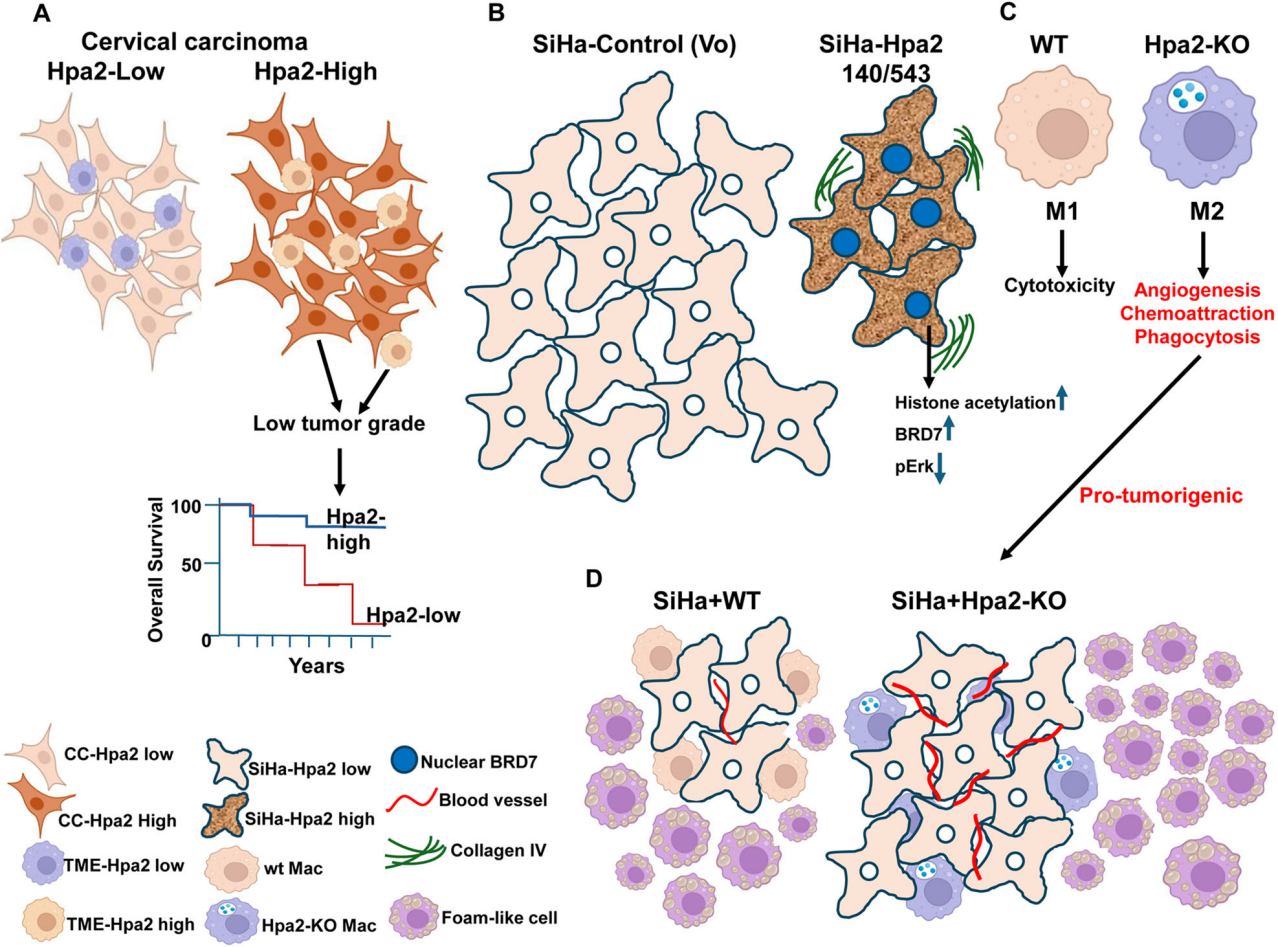
A scheme summarizing our main findings and a proposed model. **A** Clinical relevance. High levels of Hpa2 in cervical carcinoma (CC) lesions, or in cells that comprise the tumor microenvironment (TME) are associated with low tumor grade and good prognosis. **B** Experimental approaches. Overexpression on Hpa2 and the 140/543 mutants in SiHa cervical carcinoma cells results in smaller tumors associated with lower levels of Erk phosphorylation, and increased incidence of nuclear BRD7 and histone acetylation on lysine 9. **C** Macrophage polarization. In the absence of Hpa2 (Hpa2-KO), macrophages are shifted towards M2 polarization, exerting increased phagocytic, chemoattraction, and angiogenic capacities. **D** Co-implantation. Co-injection of SiHa cells together with Hpa2-KO macrophages resulted in bigger and more vascularized tumors vs tumors produced by SiHa cells implanted with control (wt) macrophages. Interestingly, foam-like macrophages were noted to accumulate adjacent to the tumor mass. Given the pro-tumorigenic properties of foam cells, this should be kept in mind in approaches utilizing macrophages as an anti-cancer tool.

Hpa2 is detected in the normal epithelium of the cervix, but its expression was decreased substantially in cervical carcinoma, where 43% of the cases were scored as Hpa2-negative. This expression pattern was noted previously in bladder, gastric, hepatocellular and breast carcinomas [13, 15, 16, 20] and is thought to reflect the expression pattern of tumor suppressors. Moreover, most tumors that retained high levels of Hpa2 were categorized as low grade, a pathological evaluation of the differentiation state of the tumor cells. Association between Hpa2 levels and low tumor grade was found previously in other types of carcinomas [14, 16, 18, 20], suggesting that Hpa2 functions to maintain epithelial cell differentiation. This is also supported by the induction of cytokeratins that depict epithelial cells [19, 21]. Interestingly, we found that Hpa2 staining is not restricted to the tumor cells, and cells in the tumor microenvironment are also stained positive for Hpa2 (Figs. 1D and 8A). Scoring the intensity of Hpa2 staining in the tumor microenvironment, most likely immune cells, revealed an inverse correlation with tumor grade (i.e., high levels of Hpa2 associates with low tumor grade; Table 2). A recent study gathered data of 5591 cervical carcinoma patients from the Surveillance, Epidemiology, and End Results (SEER) database, and examined parameters that affect patient survival. Notably, a multivariate Cox regression analysis indicated that, among other parameters, tumor grade is an independent prognostic factor affecting survival [24]. Other studies support the significance of tumor grade as a prognostic measure for cervical cancer [23, 25]. This led us to conclude that both tumor- and host-derived Hpa2 affect the prognosis of cervical carcinoma patients (Fig. 8A). This finding significantly expands the scope of Hpa2 function in tumorigenesis and implies that the presence, rather than the source, of the secreted Hpa2 protein is potentially very important in cervical cancer and possibly other types of cancer. This may open new concepts for Hpa2-based therapeutics, administrating patients with purified Hpa2 or Hpa2-derived fragments and peptides. Hpa2-based therapy is supported by the ability of recombinant Hpa2 or Hpa2-derived peptides to protect against conditions of sepsis and LPS-induced renal failure [10, 11]. The capacity of therapeutic plasma exchange (TPE) to protect the vascular glycocalyx in patients with early septic shock implies that the secreted Hpa2 reaches the circulation in a functional state and can replace the endogenous Hpa2 that is downregulated in sepsis [46]. Implementation of Hpa2-based therapy in tumorigenesis awaits in-depth investigation.

The function of Hpa2 in cervical cancer was further examined in a tumor model in mice. We found that overexpression of Hpa2 in SiHa and HeLa cells attenuates tumor growth, in agreement with a similar function of Hpa2 found in several other carcinoma and sarcoma models [16, 17, 19, 21, 22]. Notably, even more potent inhibition of tumor growth was executed by cells overexpressing the 140 and 543 mutants of Hpa2 (Figs. 1B and 8B). This was surprising, given that these missense mutants originate from families exhibiting UFS, a rare autosomal recessive disease featuring urinary voiding dysfunction [29, 38], and thus expected to exert Hpa2-null phenotype. These mutants were also more effective than WT Hpa2 in several other features. For example, attenuation of Erk phosphorylation and FSP1/S100A4 expression were evidently more effective by the 140 and 543 mutants, whereas syndecan-1, tumor fibrosis (i.e., collagen deposition), and deposition of collagen IV, were increased by the 140/543 mutants vs WT Hpa2. This, and the increased recruitment of macrophages by Hpa2 and the 140/543 mutants indicates that Hpa2 shapes the tumor microenvironment.

High-affinity interaction with HS is an important feature of Hpa2 [7] and is thought to underlie the function of Hpa2 in cell adhesion and migration. This was concluded because exogenous addition of Hpa2 was noted to attenuate cell migration and this effect was prevented by heparin [47]. However, since the 543 mutant failed to bind heparin [29] (Supplementary Fig. 1C), it appears that in cervical cancer, this mutant and possibly WT Hpa2, function in HS-independent manner. Since the 543 mutant does not get secreted [29] (Supplementary Fig. 1C), its ability to attenuate tumor growth is most likely excreted from within cells. A possible explanation for the superb function of the 140 and 543 Hpa2 mutants in SiHa tumor model is conceivably connected to their cellular localization. As expected, WT Hpa2 preferentially localizes to the cell cytoplasm. Given that Hpa2 is a secreted protein, this ‘cytoplasmic’ staining mostly reflects its biosynthetic route and marks the ER and Golgi apparatuses. In striking contrast, the 140/543 mutant forms mostly assumed nuclear localization (Fig. 3B, left). A link between nuclear localization of Hpa2 and decreased tumor growth was reported in sarcoma [22]. More recently, Hpa2 was noted to preferentially localize to the nuclei of normal breast epithelium but lose its nuclear localization in breast carcinoma [27]. Notably, tumor growth was noticeably attenuated once Hpa2 was directed to the cell nucleus (via NLS) of breast carcinoma cells [27]. Together, these results strongly imply that in certain malignancies, Hpa2 functions in the cell nucleus to restrain tumor growth. The Pro140Arg and Asn543Ile point mutations likely resulted in a mild change in the Hpa2 protein conformation that favors its interaction with a shuttle protein that directs Hpa2 to the cell nucleus.

We identified BRD7 as a Hpa2-binding protein and a candidate shuttle protein for Hpa2. Moreover, we found increased levels of BRD7 in tumors produced by Hpa2 and the 140/543 mutants (Figs. 3B, 8B and Supplementary Figs. 3G and 4E). These findings open a new perspective on the molecular mechanism underlying the anti-tumorigenic properties of Hpa2. The expression of BRD7 is downregulated or lost in breast, ovarian, pancreatic, nasopharyngeal, and colorectal carcinomas, as well as in lung adenocarcinoma and osteosarcoma [40, 48], an expression pattern that is also described for Hpa2 [49] and is typical for tumor suppressors. Indeed, BRD7 is recognized as a tumor suppressor in several types of cancer [40, 48]; in breast cancer, the survival of patients exhibiting nuclear BRD7 is significantly prolonged vs patients showing cytoplasmic BRD7 [50]. Furthermore, BRD7 is reported to attenuate Erk signaling, induce the expression of the pro-apoptotic protein Bax, and is also implicated in the VEGF pathway [40, 51, 52], thus mimicking molecular determinants that were affected by Hpa2 and the 140/543 mutants (Figs. 2 and 3). We conclude that Hpa2 induces the expression and nuclear localization of a strong tumor suppressor such as BRD7, while BRD7 can interact with Hpa2 and shuttle it to the cell nucleus. BRD7, alone or in complex with Hpa2, binds to acetylated histone 3 and affects gene transcription related to various biological processes, ranging from embryonic development [53] to tumor progression.

As noted previously, overexpression of Hpa2 facilitated conditions of stress exemplified by increased phosphorylation of p38, the stress arm of the MAPK pathway (Supplementary Fig. 4A, right). Interestingly, expression of HIF1α and expression of genes under HIF1α transcriptional regulation such as VEGF-A and CAIX were decreased markedly in tumors produced by cells overexpressing Hpa2 or the 140/543 mutants (Fig. 2). Notably, CAIX is considered pro-tumorigenic and inhibitors of CAIX represent new therapeutic strategy in cervical cancer [54, 55]. Decreased expression of CAIX and the pro-angiogenic mediator VEGF-A are thus likely to attenuate the growth of Hpa2 tumors. Interestingly, conditions of hypoxia were noted to facilitate the transition of macrophages toward a pro-tumorigenic, M2 state in cervical cancer [56]. Lower levels of hypoxic conditions in Hpa2 tumors thus offer an additional route by which Hpa2 shapes the tumor microenvironment, affecting macrophage polarization.

### Hpa2 and macrophage polarization

Macrophage polarization refers to the process by which macrophages produce distinct functional phenotypes in response to external stimuli. Macrophages can be polarized into classically activated (M1) and alternatively activated (M2) macrophages. These macrophages differ in their cell surface markers, secreted cytokines, and biological functions. Macrophage polarization is considered crucial for tissue repair and homeostasis, contributing to infection prevention, angiogenesis and immunomodulation. Although tumor-associated macrophages (TAMs) do not completely follow the M1 and M2 subtypes, they are in general thought to exhibit M2-like phenotype and facilitate tumor growth, orchestrating a wide range of pro-tumorigenic features [57–60]. Importantly, high levels of M2 macrophages in the tumor microenvironment are associated with poor prognosis of cancer patients [61], including patients diagnosed with cervical cancer [62–64]. Interestingly, we found that Hpa2 plays a role in macrophage polarization; in the absence of Hpa2, macrophages are shifted from M1 toward M2 phenotype. This is concluded based on molecular and functional characterization. For example, Hpa2-KO macrophages show a higher percentage of CD206-positive cells and express higher levels of arginase typical of M2 phenotype, while the percentage of CD11c-positive macrophages and expression of cytokines (i.e., IL-12a) and cell surface markers (i.e., CD40, CD86, MHC-II) typical of M1 macrophages are decreased in Hpa2-KO macrophages. Importantly, this molecular characterization is supported by functional assays. Hence, Hpa2-KO macrophages exhibit far greater phagocytic activity typical of M2 polarization vs WT macrophages (Fig. 4) and exhibit reduced cytotoxicity (LDH; Figs. 5B and 8C). In addition, Hpa2-KO macrophages practically failed to penetrate tumor spheroids, whereas WT macrophages seem to penetrate and dissociate these spheroids (Fig. 5A). Furthermore, once implanted in Matrigel, Hpa2-KO macrophages stimulated a strong angiogenic response within and surrounding the Matrigel plug (Fig. 6), altogether pointing to pro-tumorigenic properties of Hpa2-KO macrophages (Fig. 8C). This is best demonstrated by co-implantation of SiHa cervical carcinoma cells together with peritoneal macrophages. Importantly, co-implantation of SiHa cells and Hpa2-KO macrophages resulted in two-fold bigger tumors vs SiHa cells implanted together with WT macrophages (Fig. 8D). Inoculation of Hpa2-KO macrophages together with SiHa cells resulted in not only highly vascularized tumors but also the occurrence of tumor cells within vessels (Fig. 7A, arrow). Such intravasation of tumor cells was not observed in tumors produced by SiHa+WT macrophages or in tumors produced by SiHa cells, suggesting that the presence of Hpa2-KO macrophages converts the tumor into a more aggressive form. This is also supported by the failure of SiHa+Hpa2-KO tumors to recruit NK cells while recruiting CD206-positive macrophages to the tumor lesion. Such communication between cells that populate the tumor mass and host cells is mediated by cytokines and chemokines that recruit, or repulse, host cells to the tumor lesion. We demonstrated, for example, the induction of IL-6 and IL-10 by Hpa2-KO macrophages, cytokines that are considered pro-tumorigenic [65–67]. The full repertoire of mediators affected by the deficiency of Hpa2 is yet to be explored. Unexpectedly, inoculating macrophages together with SiHa cells not only affected the tumor microenvironment but also the massive recruitment of cells to the tumor surrounding (Fig. 7C). In morphology, these cells resembled foam cells and were stained positive for the scavenger receptor CD36, thought to play a key role in the uptake of oxidized LDL by macrophages [68], and galectin-3 implicated in lipid endocytosis [43] (Figs. 7 and 8D). Remarkably, the foam-like cells adjacent to tumors produced by SiHa+Hpa2-KO macrophages lost the F4/80 cell surface marker typical of macrophages but showed increased staining for CD206 (Fig. 7), thus resembling the characterization of Hpa2-KO macrophages. Mostly implicated in vascular biology and atherosclerosis, foam-like cells are also implicated in cancer progression and are thought to display pro-tumoral activity, possibly evolving increased production of pro-inflammatory cytokines and reactive oxygen species (ROS) [69]. The unexpected massive recruitment of foam-like cells to the tumor periphery following co-implantation of wt and Hpa2-KO macrophages with SiHa cells should be kept in mind when considering macrophages as an antitumor therapeutic approach [70].

Taken together, our results clearly implicate Hpa2 in macrophage biology. More specifically, Hpa2 is shown here to play a critical role in macrophage polarization, thus indicating that Hpa2 functions also in the tumor microenvironment (TME). In both tumor cells and cells that comprise the TME, Hpa2 exerts anti-tumorigenic features (Fig. 8A). These results support, and further expand, the notion that Hpa2 functions as a tumor suppressor and highlights BRD7 as a molecular determinant underlying its tumor-suppressing properties.

## Supplementary information

Suppl. Figure legends

Suppl. Figure 1

Suppl. Figure 2

Suppl. Figure 3

Suppl. Figure 4

Suppl. Figure 5

Suppl. Figure 6

Suppl. Table 1

Suppl. Table 2

Suppl. Table 3

Suppl. Table 4

Original Data
