# Correction: Tumor- and host-derived heparanase-2 (Hpa2) attenuates tumorigenicity: role of Hpa2 in macrophage polarization and BRD7 nuclear localization

**DOI:** 10.1038/s41419-025-07380-y

**Published:** 2025-02-13

**Authors:** Soaad Soboh, Avital Vorontsova, Malik Farhoud, Uri Barash, Inna Naroditsky, Miriam Gross-Cohen, Marina Weissmann, Yasuhiko Nishioka, Adrian S. Woolf, Neil A. Roberts, Yuval Shaked, Neta Ilan, Israel Vlodavsky

**Affiliations:** 1 Technion Integrated Cancer Center, Rappaport Faculty of Medicine, Technion, Haifa, Israel; 2 Department of Cell Biology and Cancer Science, Rappaport Faculty of Medicine, Technion, Haifa, Israel; 3 Departments of Pathology, Rambam Health Care Campus, Haifa, Israel; 4 Department of Respiratory Medicine and Rheumatology, Tokushima University, Tokushima, Japan; 5 Division of Cell Matrix Biology and Regenerative Medicine, School of Biological Sciences, Faculty of Biology Medicine and Health, University of Manchester, Manchester, UK

**Keywords:** Cancer microenvironment, Cancer microenvironment

Correction to: *Cell Death and Disease* 10.1038/s41419-024-07262-9, published online 18 December 2024

In this article the author name “Yasuhiko N” is given erroneously. It should read “Nishioka Y”.

The original article has been corrected.

