# Supplementary material for: Tumor- and host-derived heparanase-2 (Hpa2) attenuates tumorigenicity: role of Hpa2 in macrophage polarization and BRD7 nuclear localization
Source: Cell Death Dis. 2024 Dec 18;15(12):894. doi: 10.1038/s41419-024-07262-9 (PMC11655850; doi:10.1038/s41419-024-07262-9)
Supplement: Supplementary file 1 — Suppl. Figure legends [file 41419_2024_7262_MOESM1_ESM.docx]

**Suppl. Figure legends**

**Suppl. Figure 1**. Hpa2 expression, secretion, and interaction with heparin. **A**. qPCR. Total RNA was extracted from control (Vo) SiHa cells and SiHa cells overexpressing Hpa2 (WT) and the 140 and 543 mutants. Hpa2 expression was quantified by qPCR analysis applying primers specific for human Hpa2 and is presented in relation to control (Vo) cells, set arbitrarily to a value of 1, and after normalization to the expression of actin. Cell extracts were prepared from corresponding cell cultures and subjected to immunoblotting (lower panel). **B**. Tumor growth. HeLa cells were transfected with WT Hpa2 and the 140 and 543 mutants along with a control, empty vector (Vo). Cells (5x10^6^) were inoculated subcutaneously in SCID mice and tumor weight was measured after 4 weeks. **C**. Hpa2 secretion. The indicated HEK293 cells were cultured in the absence (0) or presence of the indicated concentration of heparin. Medium was collected after 24 h and subjected to immunoblotting applying anti-Hpa2 antibody. Note that the 543 mutant fails to get secreted and cannot be detected in the cell culture medium even in the presence of high concentrations of heparin.

**Suppl. Figure 2**. Hpa2 promotes breast tumor growth and metastasis. **A-D**. Triple-negative MDA-MB-231 cells. Breast carcinoma MDA-MB-231 cells were transfected with WT Hpa2 and Hpa2 mutants 140 and 543 as well as control empty vector (Vo). Following selection, cells (2.5x10^6^/50 microliter) were implanted orthotopically into the mammary gland of NOD/SCID mice (n=7 per group). After 4 weeks, when tumors reached the size of ~7x7 mm (~170 mm^3^), tumor xenografts were removed under anesthesia and weighed (**A**). Wounds were sutured, and mice were kept for additional 4 weeks to enable metastases to grow. Mice were then sacrificed and lungs were collected. **B.** 5-micron sections of formalin-fixed, paraffin-embedded lungs were subjected to H&E staining to visualize lung metastasis. Shown are representative lung images at x5 magnification. The number of lung metastases per field was counted and is shown graphically in (**C**). The area of the lungs occupied by tumor cells was analyzed by Image Pro software and is shown graphically in (**D**). **E**. Triple-negative MDA-MB-468 cells were similarly transfected with the Hpa2 gene constructs, inoculated orthotopically into the mammary gland of NOD/SCID mice (n=7 per group), and lung metastasis was quantified 4 weeks after the removal of the primary lesions. The area of the lungs occupied by tumor cells was analyzed by Image Pro software and is shown graphically.

**Suppl.** **Fig. 3**. Quantification of the immunostainings. Staining intensity (arbitrary units) is presented as mean±SE of at least 5 independent images of each of the indicated immunostaining. Unpaired two-tailed Student’s T test was used to determine the statistical significance of the staining in Hpa2 and the Hpa2 mutants 140/543 images vs control (Vo) images.

**Suppl. Figure 4**. **A**. Immunostaining. 5-micron sections of the indicted SiHa tumor xenograft were subjected to Masson's/Trichrome staining, decorating (in blue) fibrillary collagen (left panels). Sections were also subjected to immunostaining for phospho-p38 (p-p38; right panels). Original magnifications: left panels x25 (Scale bars represent 200 microns), right panels x100 (Scale bars represent 50 microns). **B**. Immunoblotting. The indicted tumor extracts were subjected to immunoblotting applying anti-CAIX (upper panel) and anti-actin (lower panel) antibodies. Note a marked decrease in CAIX expression in tumors produced by cells over-expressing wt Hpa2/140/543 vs control (Vo) cells. **C**. KM plot. A 5-year Kaplan-Meier survival estimate of cervical carcinoma patients (n-304) exhibiting low or high levels of syndecan-1, is shown. Note prolong survival of patients exhibiting high levels of syndecan-1. **D**. A schematic illustration of the Ras recruitment system. **E**. Immunostaining. 5-micron sections of tumor xenografts produced by MDA-MB-231 control (Vo) cells and cells over-expressing Hpa2 or nuclear-targeted Hpa2 (Nuc) were subjected to immunostaining applying anti-BRD7 (left) and anti-histone 3 acetylated at lysine 9 (AcH3K9; right panels). Original magnifications x100 (Scale bars represent 50 microns). Note a substantial increase in the levels of nuclear BRD7 upon nuclear targeting of Hpa2 (Nuc). **F**. Immunoblotting. Extracts of control (Vo), Hpa2 and Hpa2-Nuc MDA-MB-231 cells were subjected to immunoblotting applying anti-acetylated histone 3 on lysine 9 (AcH3(K9); upper panel) and anti-actin (lower panel) antibodies.

**Suppl. Figure 5**. Cytof and FACS analyses of bone marrow cells. Cells were collected from the bone marrow of WT and Hpa2-KO mice (n=5) and subjected to Cytof analysis, comparing the abundance of cell populations within the bone marrow (**A**). Summary of the results is presented in (**B**). To confirm the differences found by Cytof, corresponding cells were subjected to FACS analysis and cell abundance in WT and Hpa2-KO marrow was evaluated by employing cell surface markers specific for the indicated cell type (**C**).

**Suppl. Figure 6**. Cytof analyses of spleen cells. Cells were isolated from the spleen of WT and Hpa2-KO mice (n=5). Cells were subjected to Cytof analysis, comparing the abundance of cell populations within the spleen (**A**). Summary of the results is presented in (**B**). Cells were subjected to FACS analysis and cell abundance in WT and Hpa-KO spleen was evaluated by employing cell surface markers specific for the indicated cell type (**C**). **D**. Peritonitis. WT and Hpa2-KO mice (n=5) were administrated with thioglycolate as an irritant. Spleens were collected after three days, and splenocytes were subjected to FACS analyses applying the indicated cell surface markers. Note increased abundance of immune cells in the spleen of Hpa2-KO mice.
