# Supplementary figures and images for: Tumor- and host-derived heparanase-2 (Hpa2) attenuates tumorigenicity: role of Hpa2 in macrophage polarization and BRD7 nuclear localization
Source: Cell Death Dis. 2024 Dec 18;15(12):894. doi: 10.1038/s41419-024-07262-9 (PMC11655850; doi:10.1038/s41419-024-07262-9)

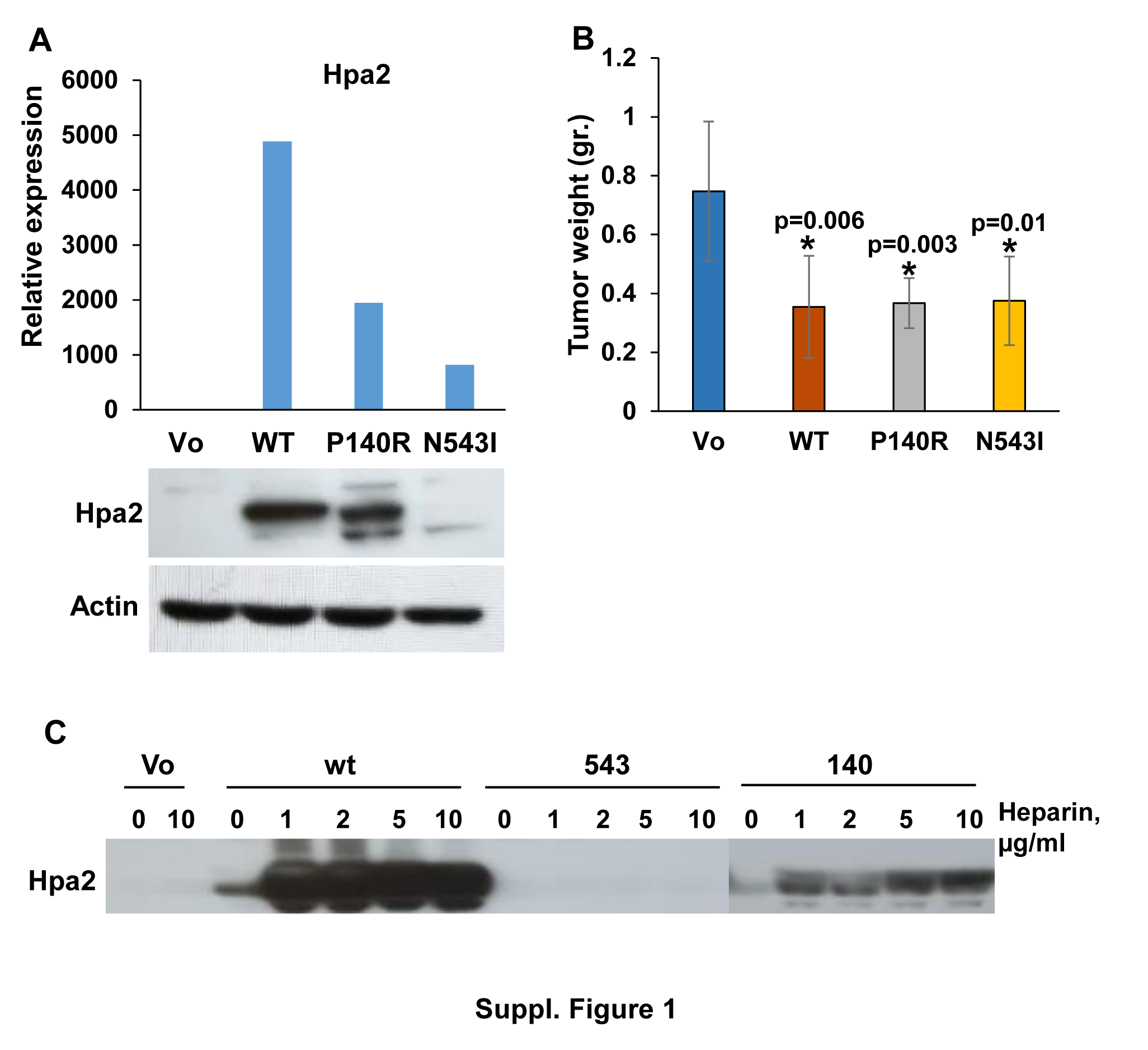

Supplement: Supplementary file 2 — Suppl. Figure 1 [file 41419_2024_7262_MOESM2_ESM.tif]

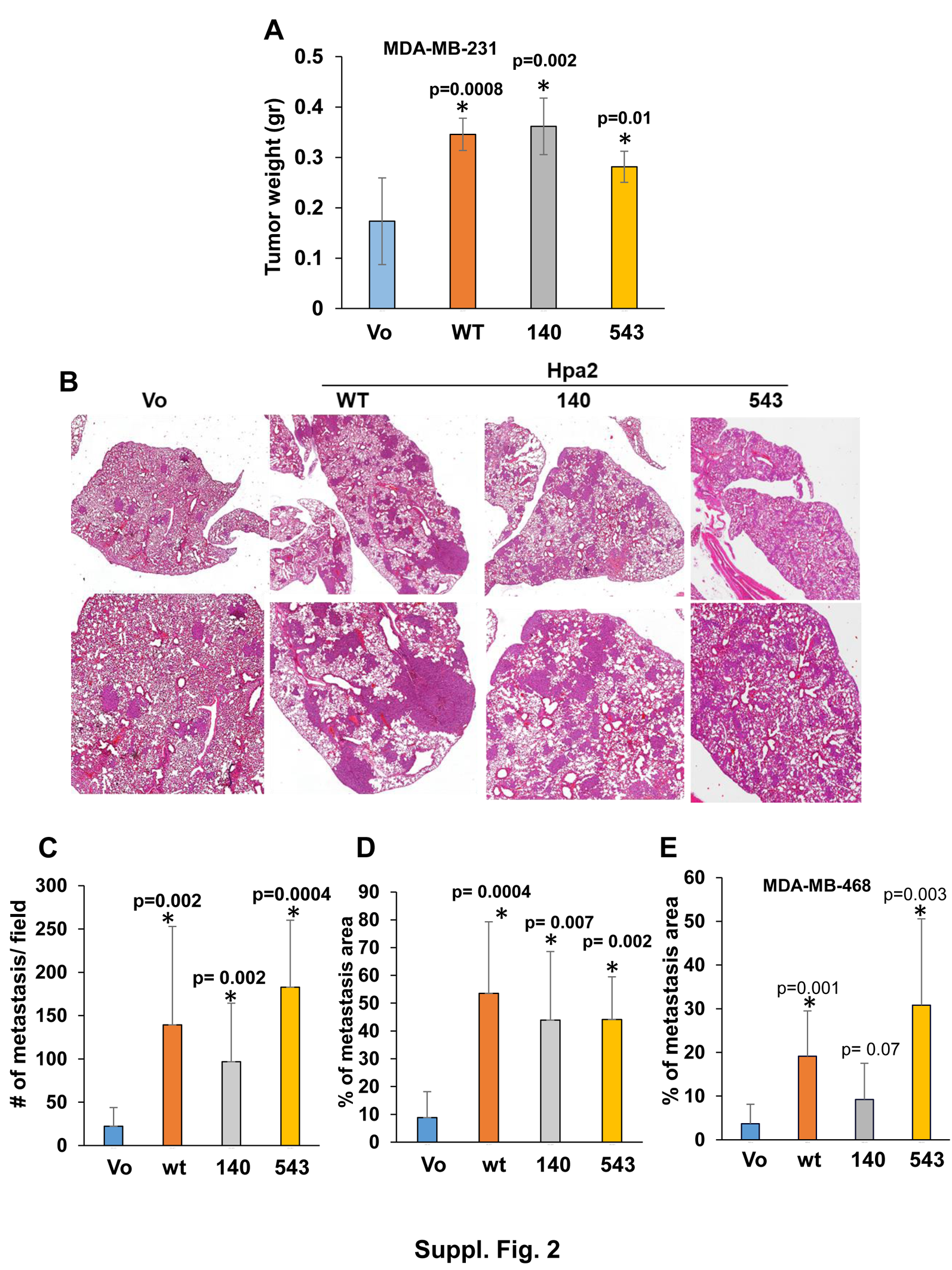

Supplement: Supplementary file 3 — Suppl. Figure 2 [file 41419_2024_7262_MOESM3_ESM.tif]

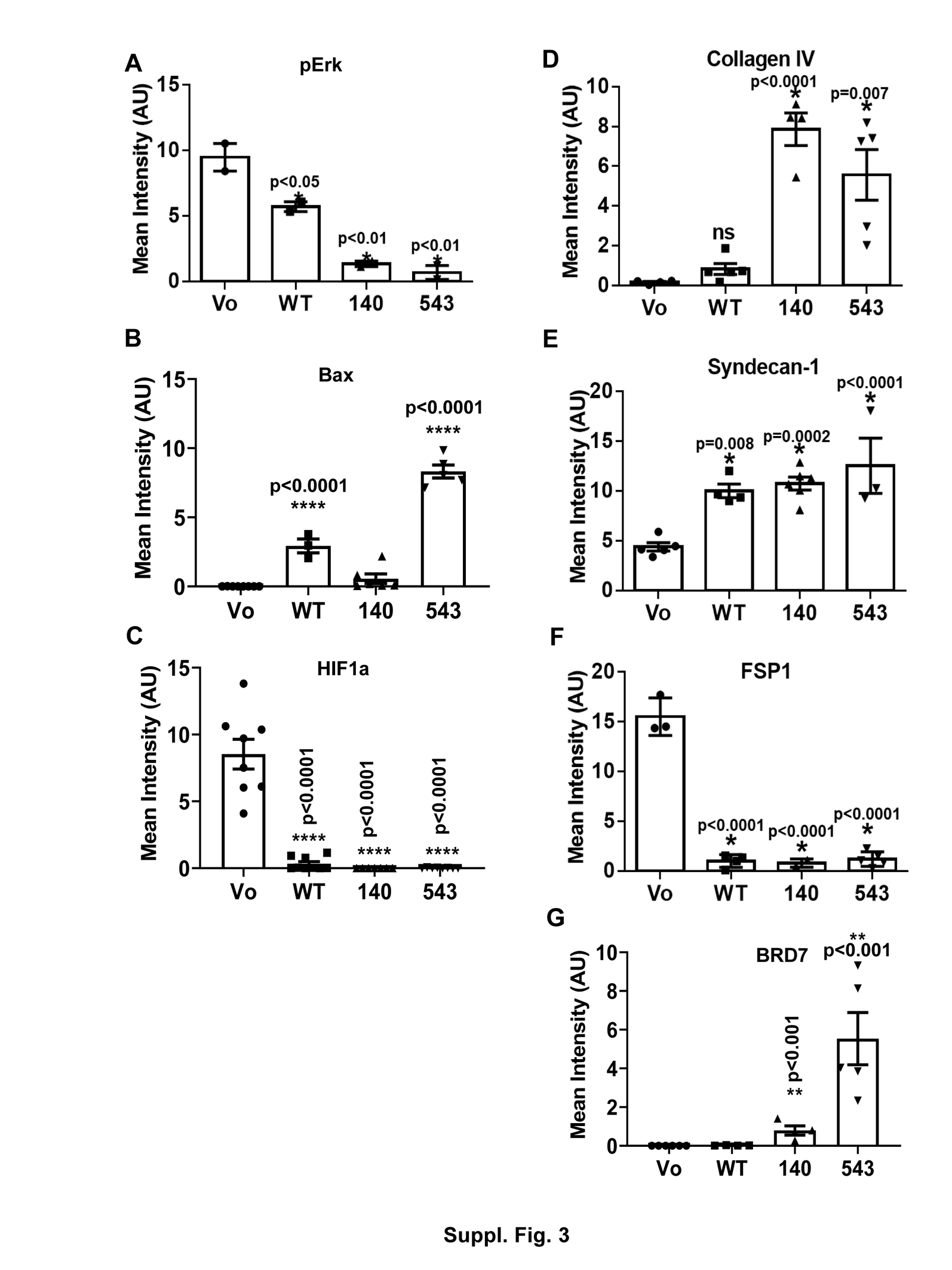

Supplement: Supplementary file 4 — Suppl. Figure 3 [file 41419_2024_7262_MOESM4_ESM.tif]

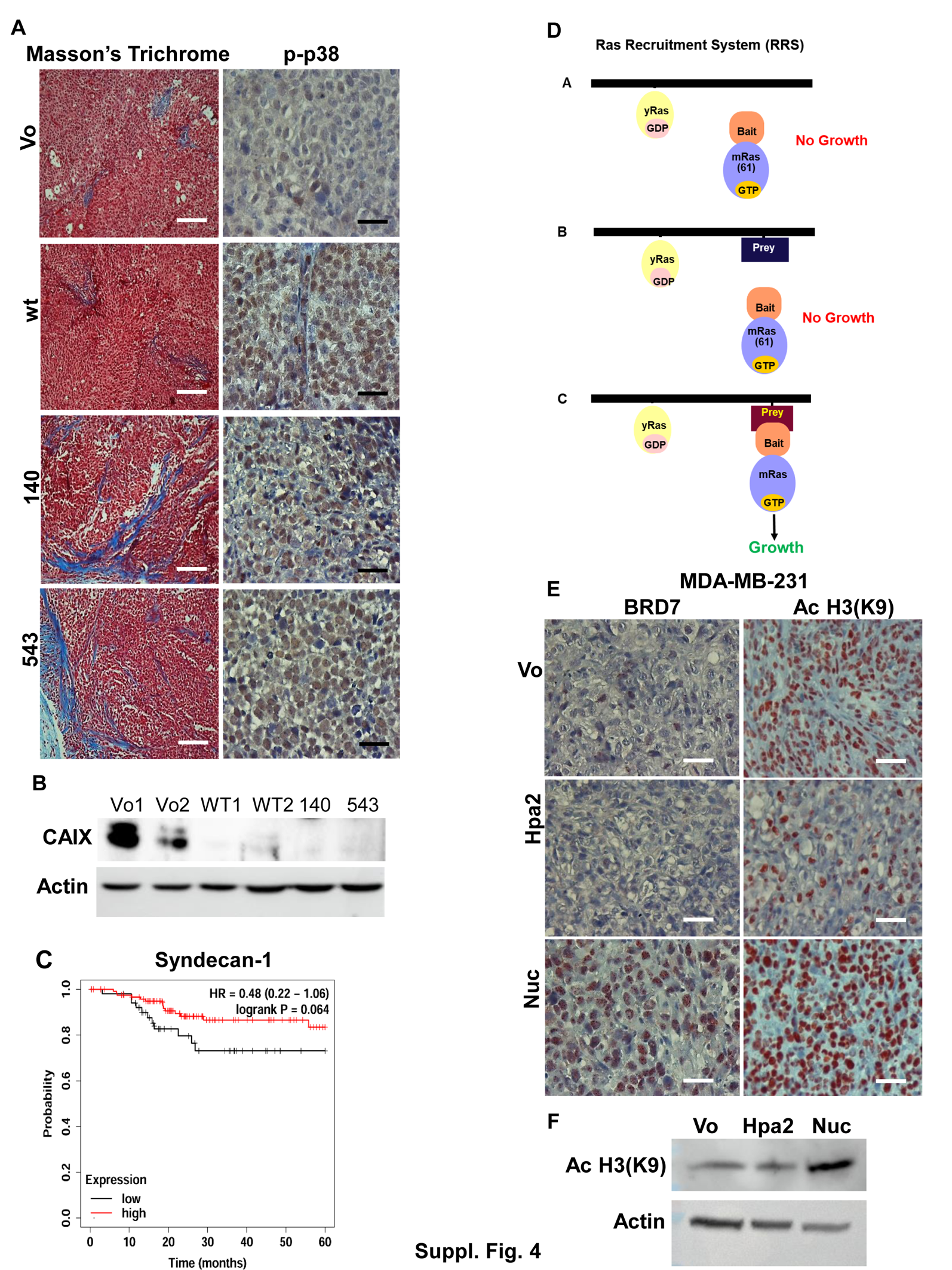

Supplement: Supplementary file 5 — Suppl. Figure 4 [file 41419_2024_7262_MOESM5_ESM.tif]

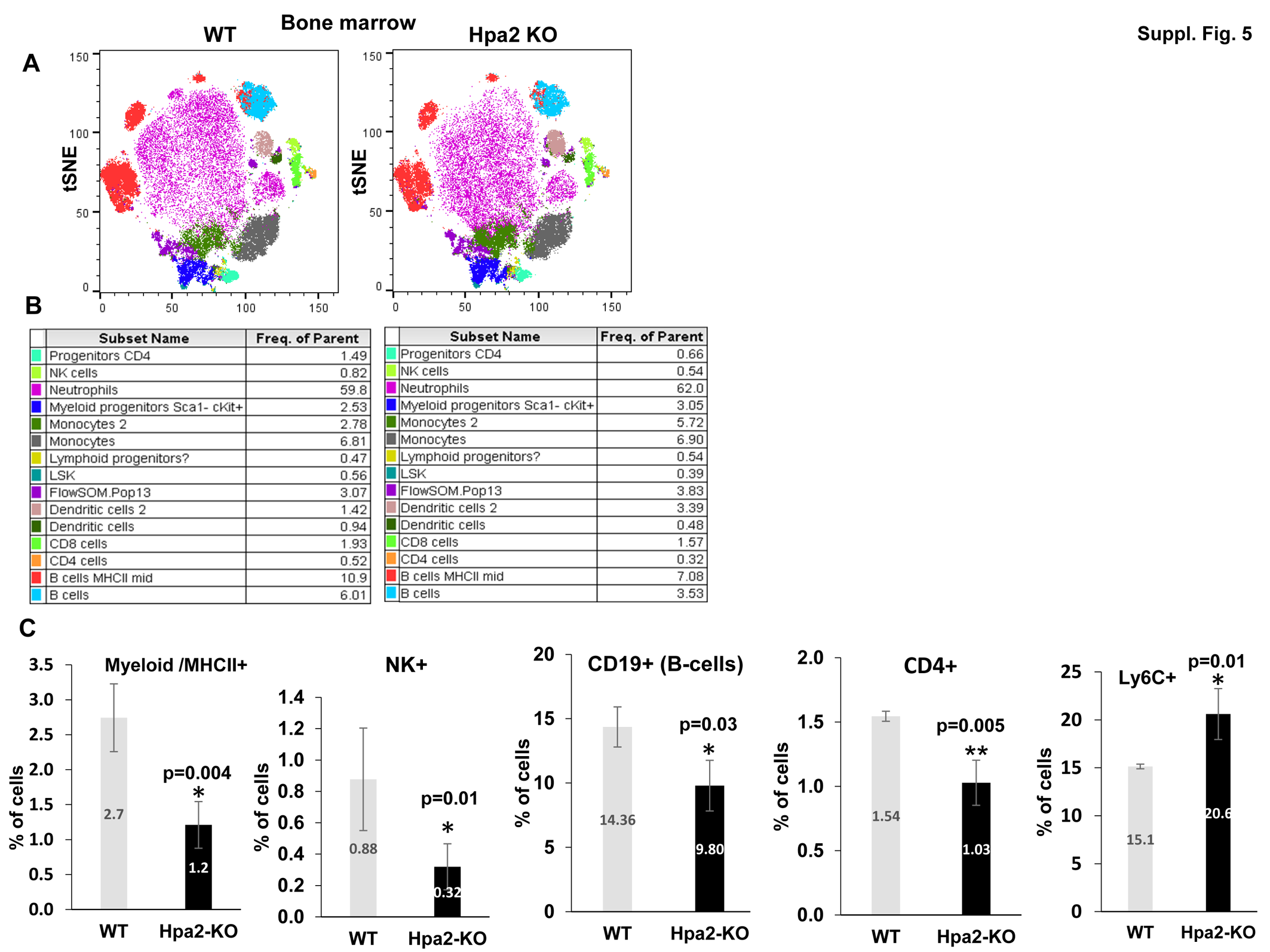

Supplement: Supplementary file 6 — Suppl. Figure 5 [file 41419_2024_7262_MOESM6_ESM.tif]

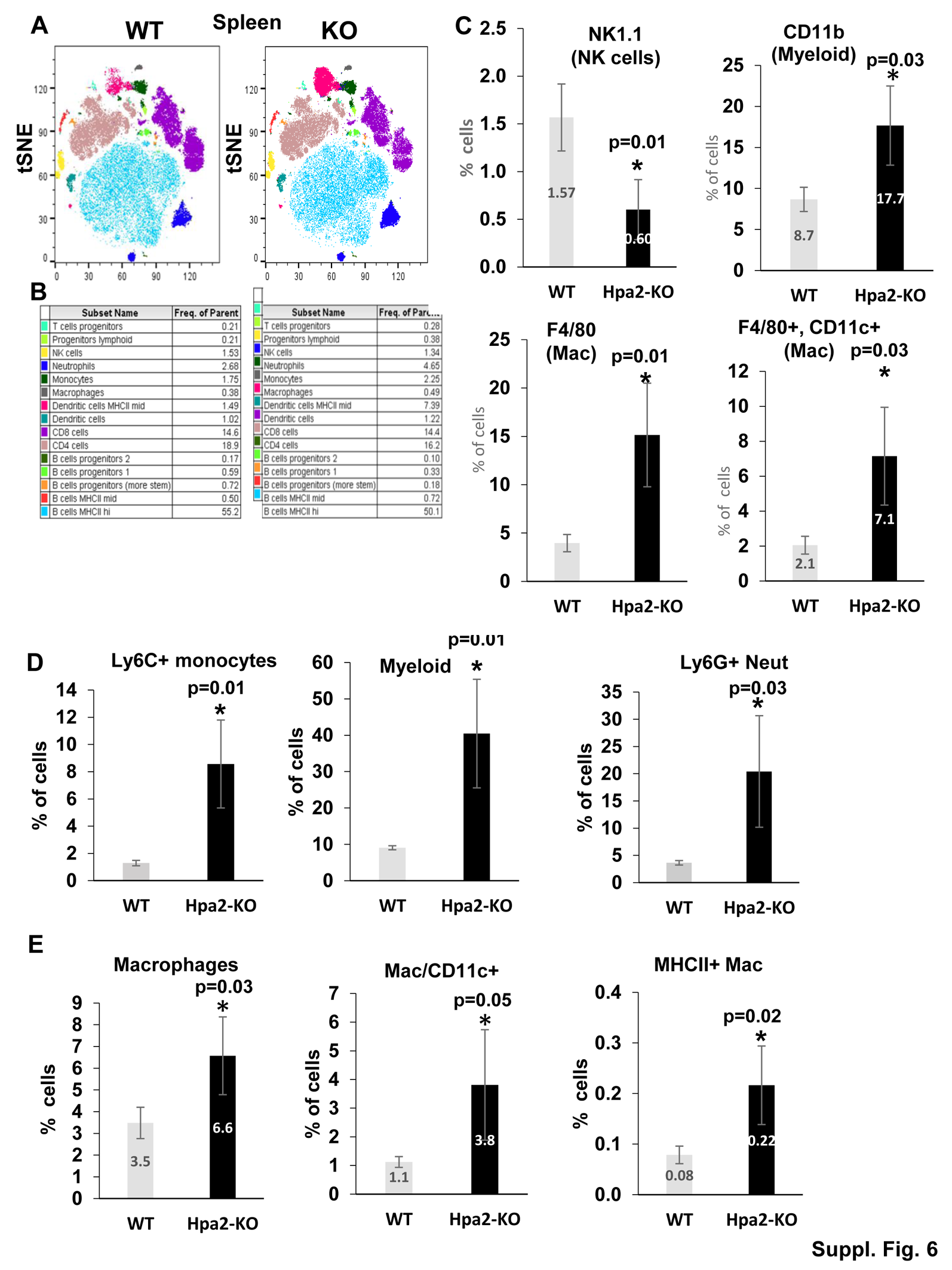

Supplement: Supplementary file 7 — Suppl. Figure 6 [file 41419_2024_7262_MOESM7_ESM.tif]

**Suppl. Table 4**. Proteins interacting with Hpa2: Ras recruitment system (RRS)


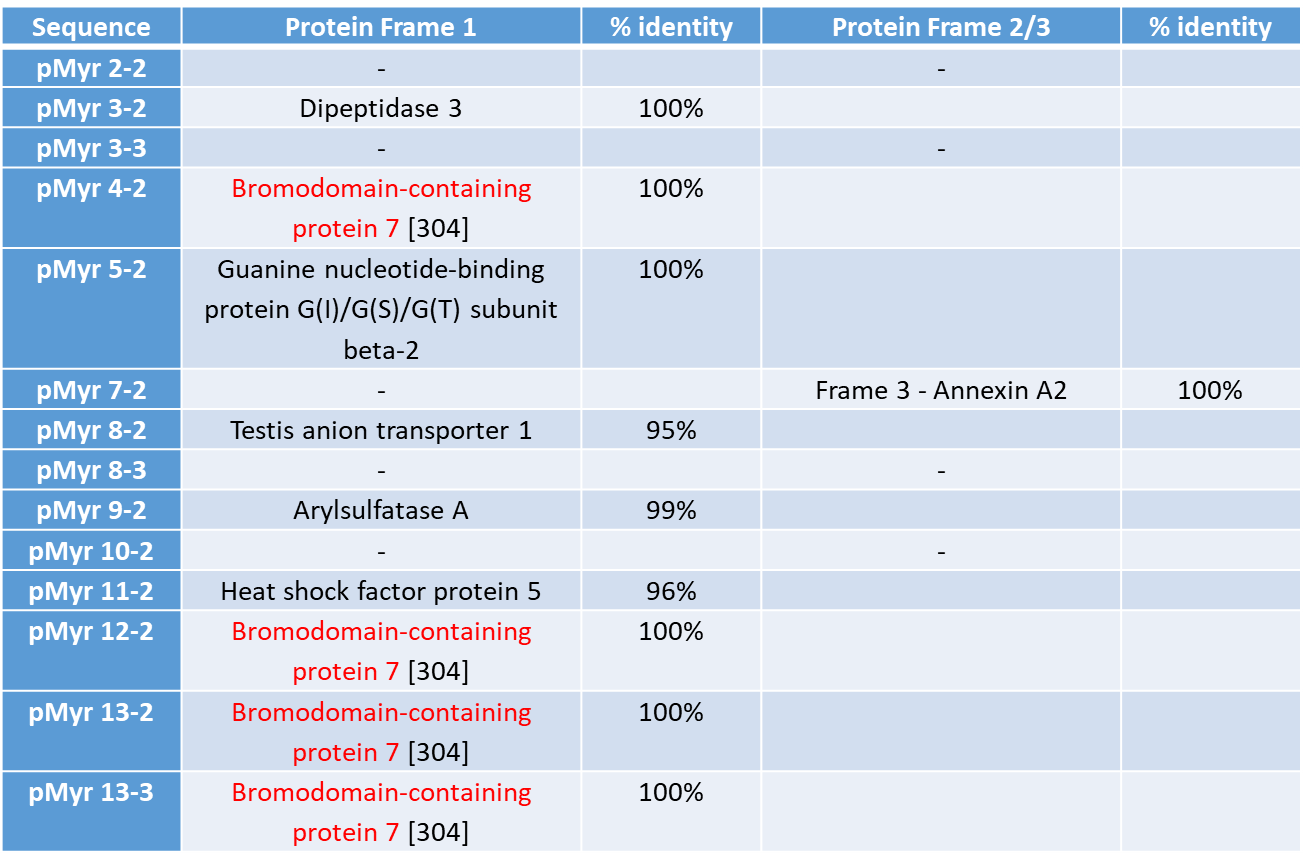

Supplement: Supplementary file 11 — Suppl. Table 4 [file 41419_2024_7262_MOESM11_ESM.docx]

13.7.21  
Soad

actin

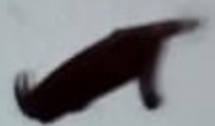  
V<sub>0</sub> WT 140 343 CM  
PC

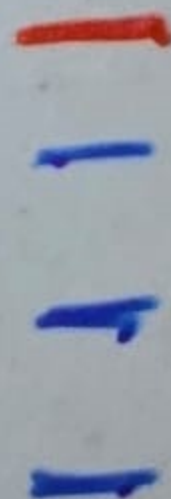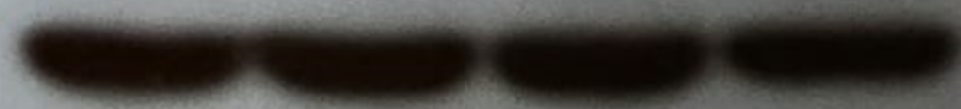

Saad Siha

Vo WT 140 543 PC

2

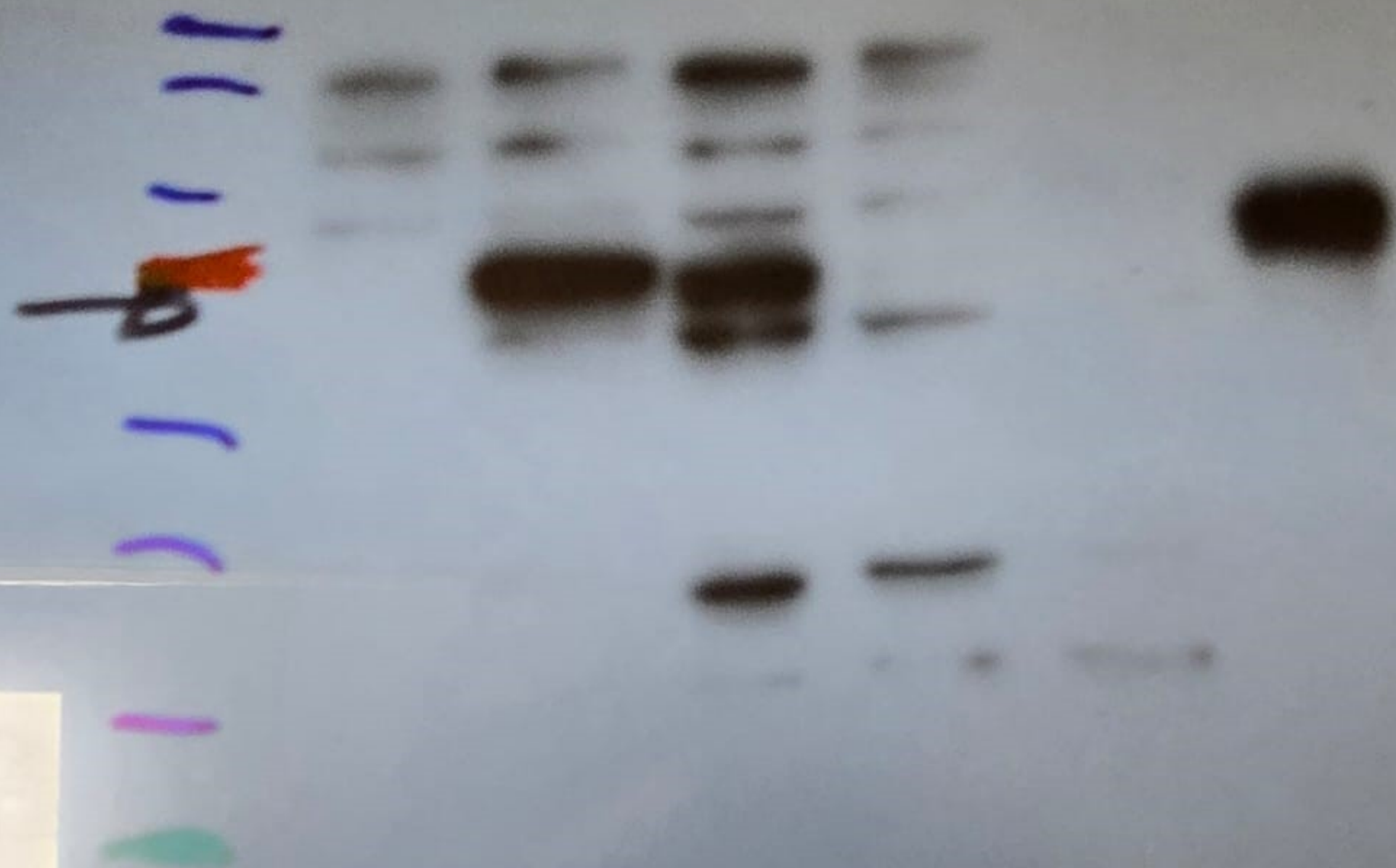

$V_0$                       WT                      543                      PC  
 10 10 1 2 5 10 1 1 2 5 10

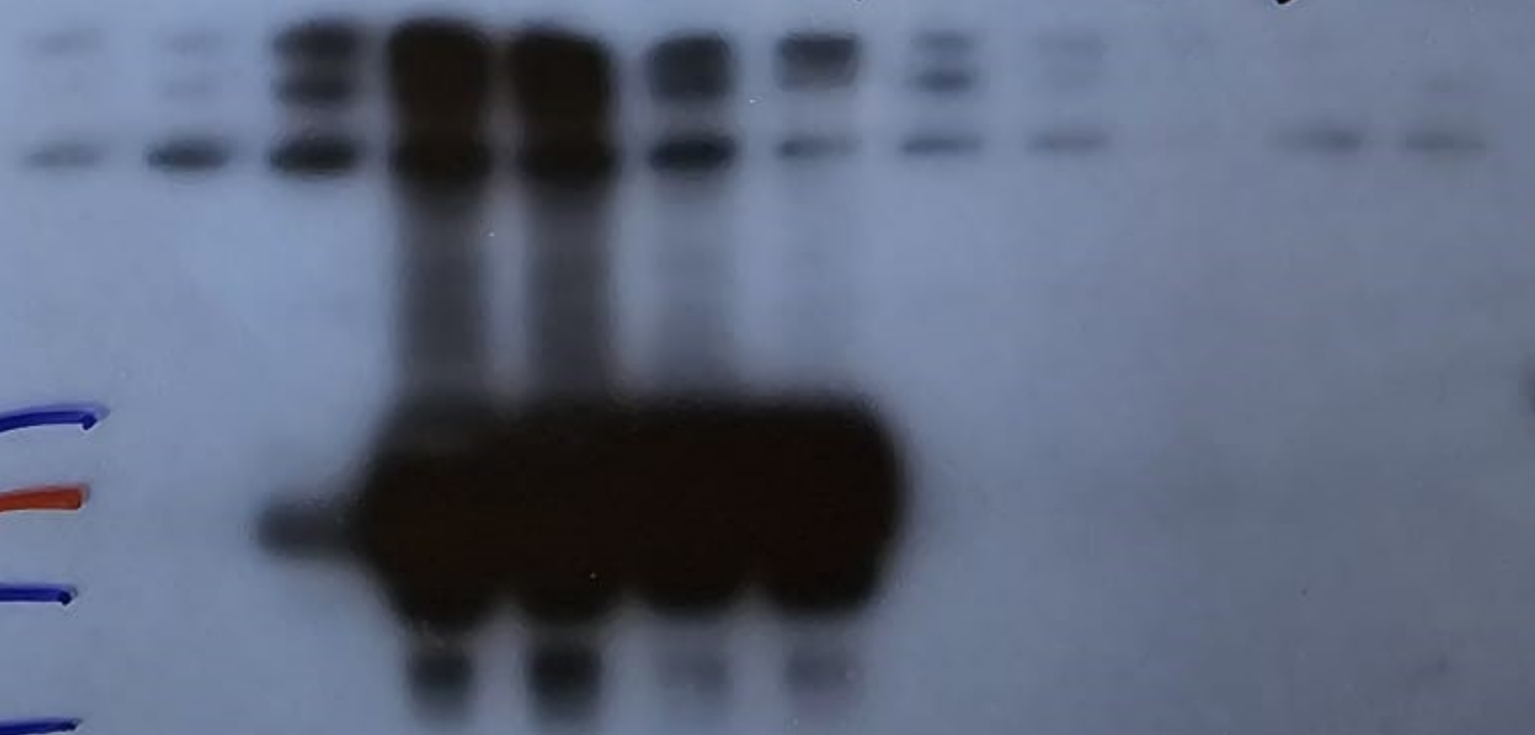

hpa2

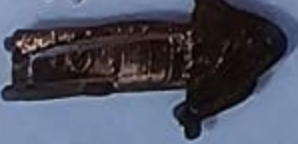

———  
 ———  
 ———  
 ———

12  
→

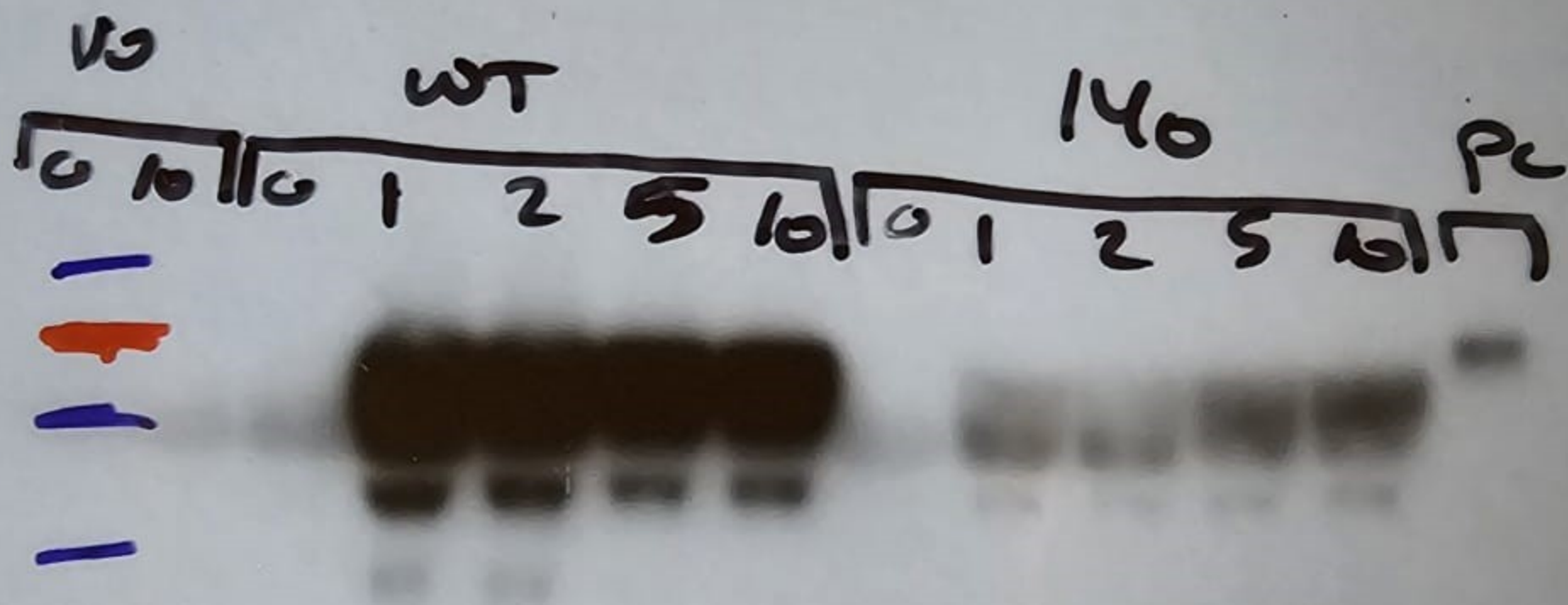

...FNU1·HRC·(2VFEELY)...

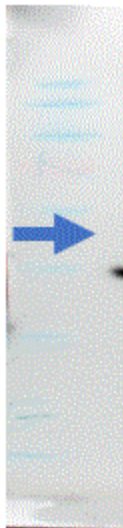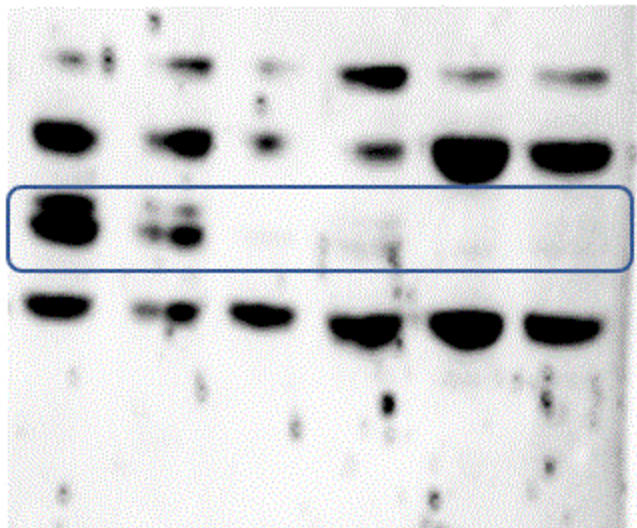

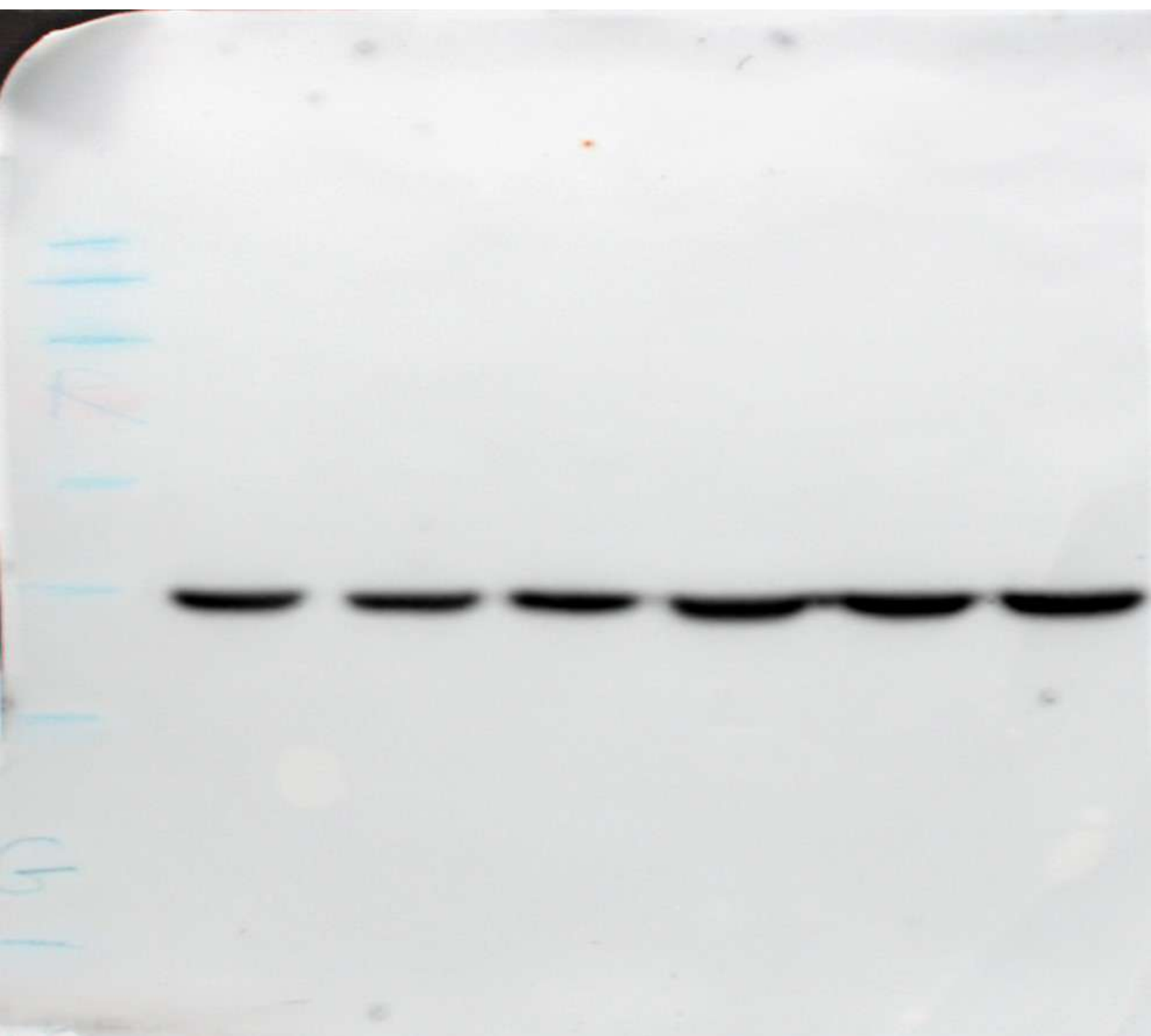



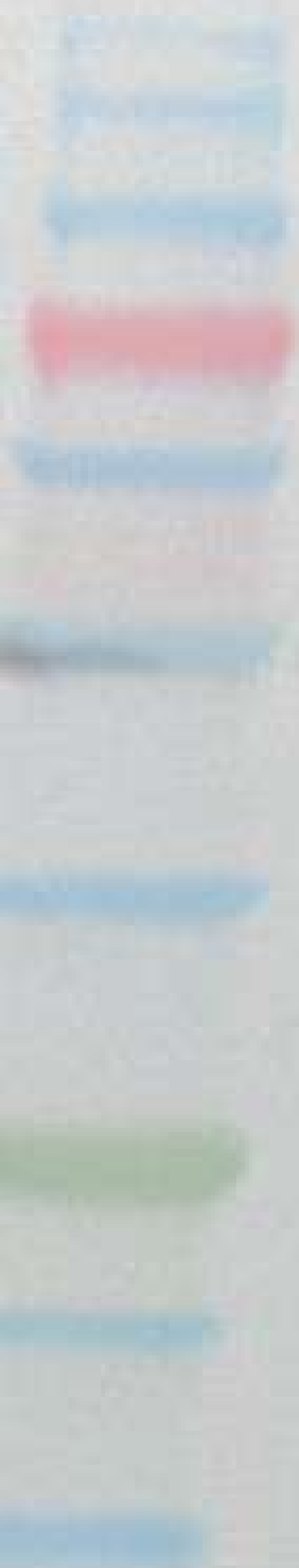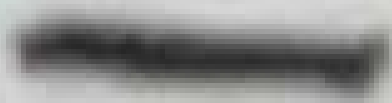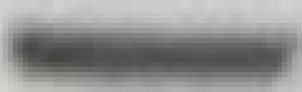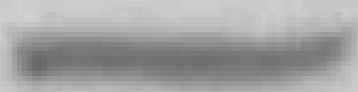

Supplement: Supplementary file 12 — Original Data [file 41419_2024_7262_MOESM12_ESM.pdf]
