# Supplementary material for: Tumor- and host-derived heparanase-2 (Hpa2) attenuates tumorigenicity: role of Hpa2 in macrophage polarization and BRD7 nuclear localization
Source: Cell Death Dis. 2024 Dec 18;15(12):894. doi: 10.1038/s41419-024-07262-9 (PMC11655850; doi:10.1038/s41419-024-07262-9)
Supplement: Supplementary file 8 — Suppl. Table 1 [file 41419_2024_7262_MOESM8_ESM.docx]

**Suppl. Table 1.** The PCR primer sets utilized in this study

| Arg-1 | F: TTGGGTGGATGCTCACACTG R: GTACACGATGTCTTTGGCAGA |
| --- | --- |
| IL12a | R: CCAGGCAACTCTCGTTCTTGT  F: AGACATCACACGGGACCAAAC |
| CD40 | F: TGTCATCTGTGAAAAGGTGGTC  R: ACTGGAGCAGCGGTGTTATG |
| CD86 | F: TCAATGGGACTGCATATCTGCC  R: GCCAAAATACTACCAGCTCACT |
| MHCII | F: AAGGCATTTCGTGTACCAGTTC  R: CCTCCCGGTTGTAGATGTATCTG |
| CD206 | F: CTCTGTTCAGCTATTGGACGC R: TGGCACTCCCAAACATAATTTGA |
| IL-6 | F: CTGCAAGAGACTTCCATCCAG  R: AGTGGTATAGACAGGTCTGTTGG |
| IL-10 | F: GCTCTTACTGACTGGCATGAG  R: CGCAGCTCTAGGAGCATGTG |
| Hpa2 | F: CCTGGAACAGTTCTAGTGCCC  R: ATGCTCCGATAGTTATTTGGCTC |
| MIP2 | F: CCAACCACCAGGCTACAGG  R: GCGTCACACTCAAGCTCTG |
| iNOS | F: TTCGGAAGGGAGCAATGCCC  R: TCTGCAGCACTTGGATCAGG |
|  |  |
