# Supplementary material for: Tumor- and host-derived heparanase-2 (Hpa2) attenuates tumorigenicity: role of Hpa2 in macrophage polarization and BRD7 nuclear localization
Source: Cell Death Dis. 2024 Dec 18;15(12):894. doi: 10.1038/s41419-024-07262-9 (PMC11655850; doi:10.1038/s41419-024-07262-9)
Supplement: Supplementary file 9 — Suppl. Table 2 [file 41419_2024_7262_MOESM9_ESM.docx]

**Suppl. Table 2**. Antibodies for cell surface markers

analyzed by flow cytometry

| **Antibody- fluorophores** | **Catalog number-** BioLegend |
| --- | --- |
| CD45(30-F11)-AF700 | 103128 |
| CD11b(M1/70)-PerCP | 101230 |
| F4/80(BM8)-PE | 123110 |
| CD206(C068C2)-BV421 | 141717 |
| CD11c(N418)-APC/Cy7 | 117324 |
| MHCII(M5/114.15.2) -BV510 | 107635 |
| Ly6C(1A8)-BV605 | 128018 |
| Ly6G(1A8)- PE/Cy7 | 127618 |
| NKp46(29A1.4)-PE/Cy7 | 137618 |
| CD19 (6D5) -PE | 115508 |
| CD4(GK1.5)-BV510 | 100449 |
