# Supplementary material for: Tumor- and host-derived heparanase-2 (Hpa2) attenuates tumorigenicity: role of Hpa2 in macrophage polarization and BRD7 nuclear localization
Source: Cell Death Dis. 2024 Dec 18;15(12):894. doi: 10.1038/s41419-024-07262-9 (PMC11655850; doi:10.1038/s41419-024-07262-9)
Supplement: Supplementary file 10 — Suppl. Table 3 [file 41419_2024_7262_MOESM10_ESM.docx]

**Suppl. Table 3**. Demographic and clinical characteristics of the patients enrolled in this study

|  |  |
| --- | --- |
| **Age** | 48.4±10.7; range=25-78 |
| **Grade**  1  2  3 | N=179*  23 (13%)  107 (60%)  49 (27%) |
| **Stage**  I  II  III | N=180  143 (79%)  34 (19%)  3 (2%) |
| **Tumor cells Hpa2:**  0  1  2 | N=178**  77 (43%)  80 (45%)  21 (12%) |
| **Immune cells Hpa2:**  0  1  2 | N=178**  53 (30%)  34 (19%)  91 (51%) |

*: information of one patient was missing

**: information of two patient was missing
